# Modulation of Aβ_1–42_ Aggregation by a SARS-CoV‑2 Protein Fragment

**DOI:** 10.1021/acs.jcim.5c01811

**Published:** 2025-10-01

**Authors:** Malinda B. Premathilaka, Ulrich H. E. Hansmann

**Affiliations:** Department of Chemistry & Biochemistry, 6187 University of Oklahoma, Norman, Oklahoma 73019, United States

## Abstract

A number of studies have pointed out the possibility that SARS-CoV-2 infections could trigger amyloid diseases such as Parkinson’s disease or type II diabetes. In the present study, we probe this question for Alzheimer’s disease, which is connected with the presence of amyloids rich in Aβ peptides. For this purpose, we study, by way of molecular dynamics simulations, the interaction between the fragment FKNIDGYFKI of the Spike protein with an Aβ_1–42_ monomer and two fibril models, one patient-derived and one synthetic. We find that the viral protein fragment appears to shift the ensemble of monomer conformations toward more aggregation-prone ones, and that fibril polymorphs found in patients with Alzheimer’s disease appear to be more stabilized than synthetic fibrils. We discuss commonalities and differences in the modulation of amyloid formation by the viral protein fragments by comparing our results with previous studies of other amyloid-forming proteins.

## Graphical abstract

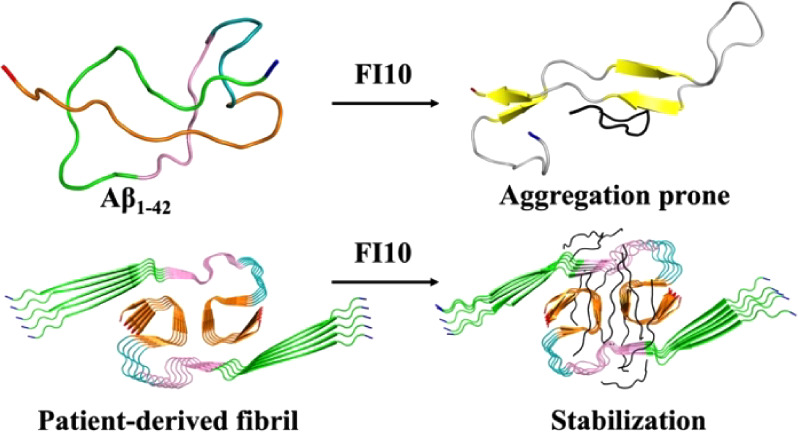

## Introduction

The presence of amyloids in humans is often associated with neurodegenerative and other diseases,^1^ but there is only an incomplete understanding of how and by what agents amyloids form and propagate into fibrils. Various studies have shown that, *in vitro,* microbial proteins can seed aggregation of Aβ and α-synuclein (αS).^2–9^ For instance, SARS-CoV-2-triggered amyloid formation is seen *in vitro* for αS^6^ which is implicated in Parkinson’s disease (PD), and correlations between COVID-19 and outbreaks of PD have been observed.^10,11^ Using extensive molecular dynamics simulations, we showed that amyloidogenic SARS-CoV-2 protein fragments can enhance aggregation of αS by shifting the ensemble of monomers toward more aggregation-prone conformations and by differentially stabilizing toxic fibril polymorphs.^12,13^ As such short fragments are cleaved from SARS-CoV-2 proteins by enzymes released from neutrophils during acute inflammation and form amyloids *in vitro*,^14^ our results point to a potential mechanism by which COVID-19 may contribute to PD.

In terms of the number of patients, a much more devastating neurodegenerative disease is Alzheimer’s disease (AD), which is connected to the presence of amyloids rich in Aβ peptides. As correlations between COVID-19 and AD appear to exist,^15^ it is possible that the presence of SARS-CoV-2 in the brain^16–18^ ultimately raises the risk for AD. This conjecture is supported by the observation that glycoproteins on herpes simplex virus 1 (HSV-1) catalyze the aggregation of Aβ.^3,19^ Similarly, the HIV-TAT protein induces Aβ amyloid formation and propagation into neurotoxic fibrils by forming complexes with Aβ.^20^ Hence, it is important to study whether and how viral proteins from SARS-CoV-2 can also seed or modulate the aggregates of Aβ proteins.

This is the purpose of the present study, where we investigate the effect of the SARS-CoV-2-specific FI10 peptide, released after cleavage by the enzyme neutrophil elastase from the spike protein (the segment 194–203: FKNIDGYFKI), on the ensemble of conformations of Aβ_1–42_ monomers. FI10 is not the only SARS-CoV-2 protein fragment that may form amyloids.^14^ For instance, Spike1058 (^1058^HGVV­FLHV­TYV^1068^), a viral fragment derived from the SARS-CoV-2 spike protein, can enhance the aggregation of Aβ_12–22_ and Aβ_27–37_ segments by increasing the β-sheet content,^21^ and our group has also worked extensively with SK9 (the 54–62 fragment of the envelope protein). In the present paper, we focus on FI10, as Nyström and Hammarström^14^ showed that this fragment is cleaved from the spike protein by an enzyme that would be released from neutrophils during inflammation. Our investigation is eased by the wealth of computational and experimental data for Aβ_1–42_ monomers and fibrils, which can help us evaluate our results. Our goal is to probe whether the monomer ensemble is shifted toward conformations that have a higher propensity for aggregation. The end product of the Aβ aggregation process is a polymorphic set of fibrils, whose structures not only depend on experimental conditions but are often correlated with the severity and progression of AD. For this reason, we also consider the effect of FI10 on the stability of two experimentally resolved fibril models: a patient-derived one with Protein Data Bank (PDB) ID 7Q4B and a synthetic fibril model with PDB ID 5KK3. Besides the potential health relevance of documenting an aggregation-modulating effect of SARS-CoV-2 proteins on Aβ_1–42_ peptides, we try to establish a general mechanism for pathogen-induced amyloid formation by comparing the results for Aβ with our earlier work studying the effect of SARS-CoV-2 protein fragments on the aggregation of αS, SAA, and amylin.^12,13,22,23^

## Materials and Methods

### System Preparation

We rely on regular molecular dynamics (MD) simulations at constant temperature and pressure to study how the ten-residue segment FKNIDGYFKI (FI10) of the Spike protein from SARS-CoV-2 affects the conformational ensemble of the Aβ_1–42_ monomer and whether the observed changes suggest an increased propensity for aggregation. While Aβ_1–40_ peptides are more common, we chose Aβ_1–42_ peptides for this investigation, as they are associated with more severe forms of AD. The initial configurations for the Aβ_42_ monomer were obtained from one of our previous works, which employed our Resolution Exchange with Tunneling (ResET) method to sample Aβ_1–42_ monomer conformations. In order to be consistent with our earlier work,^24^ we again added an acetyl and a methyl group at the N- and C-terminals, respectively.

Simulations starting from the Aβ_1–42_ monomer described above serve as a control in our study. To investigate the effect of SARS-CoV-2 protein fragments on Aβ_1–42_, we performed simulations starting from configurations where an FI10 peptide binds to this model. Following Nyström and Hammarström,^14^ we extracted the residues F194-I203 from the resolved SARS-CoV-2 Spike protein model (PDB ID 6VXX) as the initial configuration of the FI10 viral fragment, with the N- and C-terminals of the peptide capped again by NH_3_^+^ and COO^–^ groups. Using HADDOCK^25,26^ we docked the FI10 peptide in a ratio of 1:1 with the Aβ_1–42_ monomer. The resulting configurations are shown in Figure S1. Note that the FI10 peptide is not restricted to this initial position but can move freely and, over the course of the simulation, may even detach from the Aβ_1–42_ monomer.

Besides probing the interaction with Aβ_1–42_ monomers, we also looked into the effect of the viral protein fragment FI10 on fibrils formed from Aβ_1–42_ chains. Since there is considerable polymorphism in the resolved structures deposited in the Protein Data Bank (PDB), we considered in this study two distinct fibril models: the patient-derived Aβ42 fibril model of type I (PDB ID 7Q4B), which was resolved by cryo-EM for the segment of residues 9–42, and the synthetic fibril model (PDB ID 5KK3), which has been derived *in vitro* and resolved by NMR for residues 11–42. Comparing the two fibril models allows us to test whether there is a differential modulation of fibril stability by the viral protein fragment. Both fibril structures have 2-fold symmetry. The patient-derived fibril model is composed of five layers, while the synthetic fibril model is made of nine layers. To maintain consistency among the fibril simulations, we extracted the first five layers from the synthetic fibril structure for our model. In both models, the first N-terminal residues were missing, reflecting disordered N-terminal regions. We added these residues using PyMOL^27^ and capped the N- and C-terminals of the chains with acetyl and methyl groups, respectively. The so-obtained 2-fold, five-layer fibril fragments were relaxed in 3 ns simulated annealing runs, during which the experimentally resolved parts of the fibril were restrained. For this purpose, the respective fibril fragment was heated to 510 K within the first 200 ps, cooled to 310 K over the next 2600 ps, and finally equilibrated at 310 K for 200 ps. The resulting structures (shown in Figure 1e,f) served as starting configurations for our control simulations (i.e., in the absence of FI10) and were compared with simulations where ten FI10 peptides were docked with the fibril fragments (also shown in Figure S1) in a 1:1 ratio with the Aβ_1–42_ chains, using HADDOCK.^25,26^

**Figure 1:**
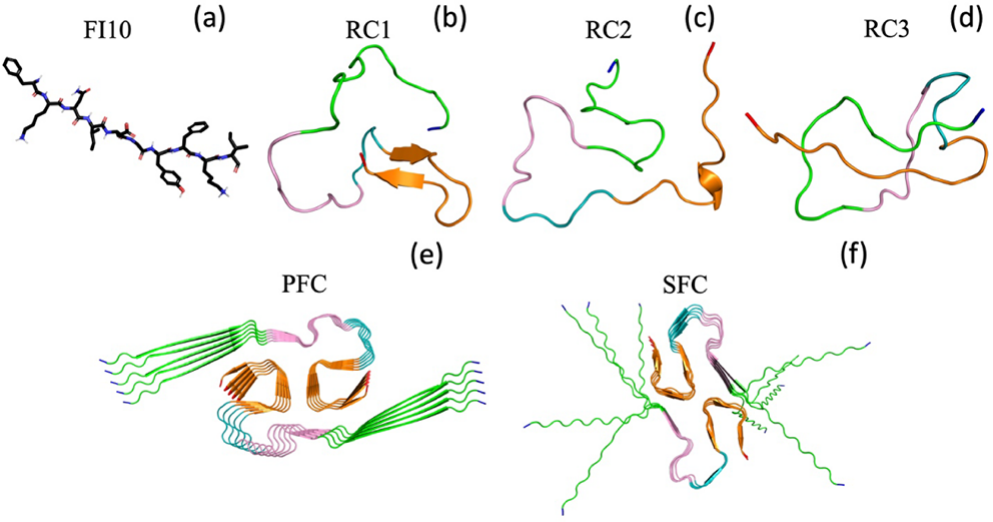
(a) Licorice representation of the SARS-CoV-2 protein fragment FI10 used in this study. Ribbon representations of the three initial configurations of Aβ_1–42_ monomers (RC1, RC2, and RC3) are shown in (b–d), with the considered regions colored as follows: green (region N, residues 1–15), pink (region M1, residues 16–24), teal (region M2, residues 25–29), and orange (region C, residues 30–42). The N- and C-termini are colored blue and red, respectively. Finally, we show sketches of the two decamer Aβ_1–42_ fibril models used in our study: the patient-derived model (PFC) with PDB ID 7Q4B (e) and the synthetic fibril model (SFC) with PDB ID 5KK3 (f).

### General Simulation Protocol

The setup and simulation of all systems rely on the GROMACS 2022 package.^28^ We use the CHARMM36m all-atom force field^29^ with TIP3P explicit water,^30^ as implemented in the GROMACS package, to describe the inter- and intramolecular interactions for the monomer and fibrils. Previous work performed in our group^31,32^ and in the literature^33–35^ has shown that this force field and water model combination is well-suited for simulations of fibrils and oligomers. Hydrogen atoms are added using the *pdb2gmx* module of the GROMACS suite.^28^ The respective starting configurations are placed in the center of the cubic box, with at least 15 Å between the solute and the edge of the box. Periodic boundary conditions are employed. The systems are solvated with water molecules, and counterions are added to neutralize the system, with Na^+^ and Cl^–^ ions at a physiological ion concentration of 150 mM NaCl. Both the number of water molecules and the total number of atoms are given in Table 1. The energy of each system is minimized by steepest descent for up to 50,000 steps, and afterward, the system is equilibrated at 310 K for 200 ps at constant volume and an additional 200 ps at constant pressure (1 bar), constraining the positions of heavy atoms with a force constant of 1000 kJ mol^–1^ nm^–2^.

**Table 1:** Simulated Systems

system description	atoms	water molecules	independent trajectories	simulation length (ns)	total sampling (ns)
Monomer Simulation					
Control					
RC1	32586	10629	1	3000	9000
RC2	29535	9614	1	3000	
RC3	27805	9038	1	3000	
With FI10					
RF1	31679	10268	1	3000	9000
RF2	33195	10772	1	3000	
RF3	31670	10265	1	3000	
Fibril Simulations					
Patient-Derived Fibril Model (PDB ID 7Q4B)					
PFC (control)	492528	161744	3	100	300
PFF (with FI10)	492424	161116	3	100	300
Synthetic Fibril Model (PDB ID 5KK3)					
SFC (control)	534894	175840	3	100	300
SFF (with FI10)	520589	170487	3	100	300

After equilibration, our monomer simulations were conducted at a constant temperature (310 K) and constant pressure (1 bar) over 3 μs for each replica. The fibril simulations were run at the same temperature and pressure but, due to the much larger system size, only for 100 ns. We used a v-rescale thermostat^36^ with a 0.1 ps coupling constant to control the temperature. The Parrinello-Rahman barostat^37^ with a 2 ps relaxation time was employed for pressure coupling. The SETTLE algorithm^38^ was used to maintain the rigidity of the molecules, while the LINCS algorithm^39^ was applied to restrain the protein bonds and keep hydrogen atoms at their equilibrium distances. In our simulations, we used a 2 fs time step to integrate the equations of motion. We employed the particle-mesh Ewald (PME) method^40^ with a 12 Å real-space cutoff and 1.6 Å Fourier grid spacing to deal with long-range electrostatic interactions. A cutoff of 12 Å was introduced to deal with short-range interactions. Table 1 shows the lengths and total sampling durations in our simulations. As discussed in the respective Results sections, the time evolution of the root-mean-square deviation of the protein and fibril structures relative to their respective starting conformations indicated that the monomer simulations converged after 1 μs, and the fibril simulations after 50 ns. Hence, only the final 2.0 μs of each monomer trajectory and the final 50 ns of each fibril trajectory were used for analysis. Atomic coordinates of the initial and final configurations of all trajectories are provided as Supporting Information.

### Trajectory Analysis

We used VMD,^41^ PyMOL,^27^ and UCSF Chimera^42^ to visualize our trajectories and conformations. GROMACS tools were employed to calculate the root-mean-square deviation (RMSD), root-mean-square fluctuation (RMSF), solvent-accessible surface area (SASA), and radius of gyration (*R*_g_). The solvent-accessible surface area was calculated using a 1.4 Å spherical probe. We performed intrastrand contact analysis using the MDTraj library^43^ and a 4.5 Å cutoff between the closest heavy atoms in a residue pair. The secondary structures of the peptides were determined using the MDAnalysis library^44^ and the Dictionary of Secondary Structure in Proteins (DSSP) algorithm.^45^ Salt bridges were analyzed using MDAnalysis with a cutoff of 4.0 Å.

We performed a cluster analysis for the monomer simulations to identify the most representative conformations during the simulation using the method explained by Daura et al.,^46^ with a cutoff of 0.5 nm for the backbone RMSD calculation in the cluster analysis. The number of contacts between FI10 and Aβ_1–42_ was calculated by using the MDAnalysis library. We used the same cutoff (4.5 Å) as for calculating the intrastrand contacts in order to calculate the contacts between Aβ_1–42_ and FI10.

We have calculated the binding free energy based on the approach described by Bellaiche and Best.^47^ This method was also previously used in a study focused on inhibiting amyloid β fibril formation,^48^ but accounting for the difference in chain lengths when calculating the concentration *C*_sim_ and approximating the binding affinity of FI10 by the sum of the binding free energies of individual residues $\Delta G=\sum \Delta G_{i}$ , with

$$\Delta G_{i}=RT\;\ln\left(\frac{K_{\mathrm{bind}}^{i}\times C_{\mathrm{sim}}}{C_{0}}\right)$$

where $K_{\mathrm{bind}}^{i}=\left(\frac{1-p_{i}}{p_{i}}\right)$ , $p_{i}$ is the binding probability of the *i*th residue, *C*_sim_ is the simulation concentration, *C*_0_ is the standard concentration of 1 M, *R* is the gas constant, and *T* is the temperature.

## Results and Discussion

### Monomer Simulations

Aβ_1–42_ is an intrinsically disordered peptide. While models of Aβ_1–42_ peptides with partially stable secondary structures have been deposited in the Protein Data Bank (for instance, the helix-rich model with PDB ID 1Z0Q), these models reflect experimental conditions that stabilize secondary structure and are not necessarily representative of the peptide in water. For this reason, we use equilibrium configurations taken after 1 μs from trajectories obtained in previous all-atom molecular dynamics simulations^24^ (see the Methods section) as starting configurations for our control simulations (without FI10 bound to it),. As expected for such equilibrium configurations, all three are disordered with little secondary structure (in R1, β-strands for segments I29-A31 and V39–I41; in R2, a short helical segment L34-V36, and only a coil structure in R3).

Over the course of the trajectories, quantities such as the radius of gyration, solvent-accessible surface area, or number of intrastrand contacts fluctuate around an equilibrium value (see Figure 2a–c. However, it is not obvious that these three initial configurations also represent equilibrium structures for the complex formed by Aβ_1–42_ with FI10, and some time may be needed to approach equilibrium. For this reason, we chose for our analysis only the last 2 μs of our trajectories, at which time the root-mean-square deviations from the start (not shown) had reached a plateau for all systems. As observed similarly in previous work for αS, the presence of the viral protein fragment FI10 shifts the ensemble of Aβ_1–42_ monomer configurations to more stretched and potentially more aggregation-prone ones, with a larger radius of gyration (*R*_g_) and solvent-accessible surface area (SASA), but a lower number of intrastrand contacts (see Figure 2d–f).

**Figure 2:**
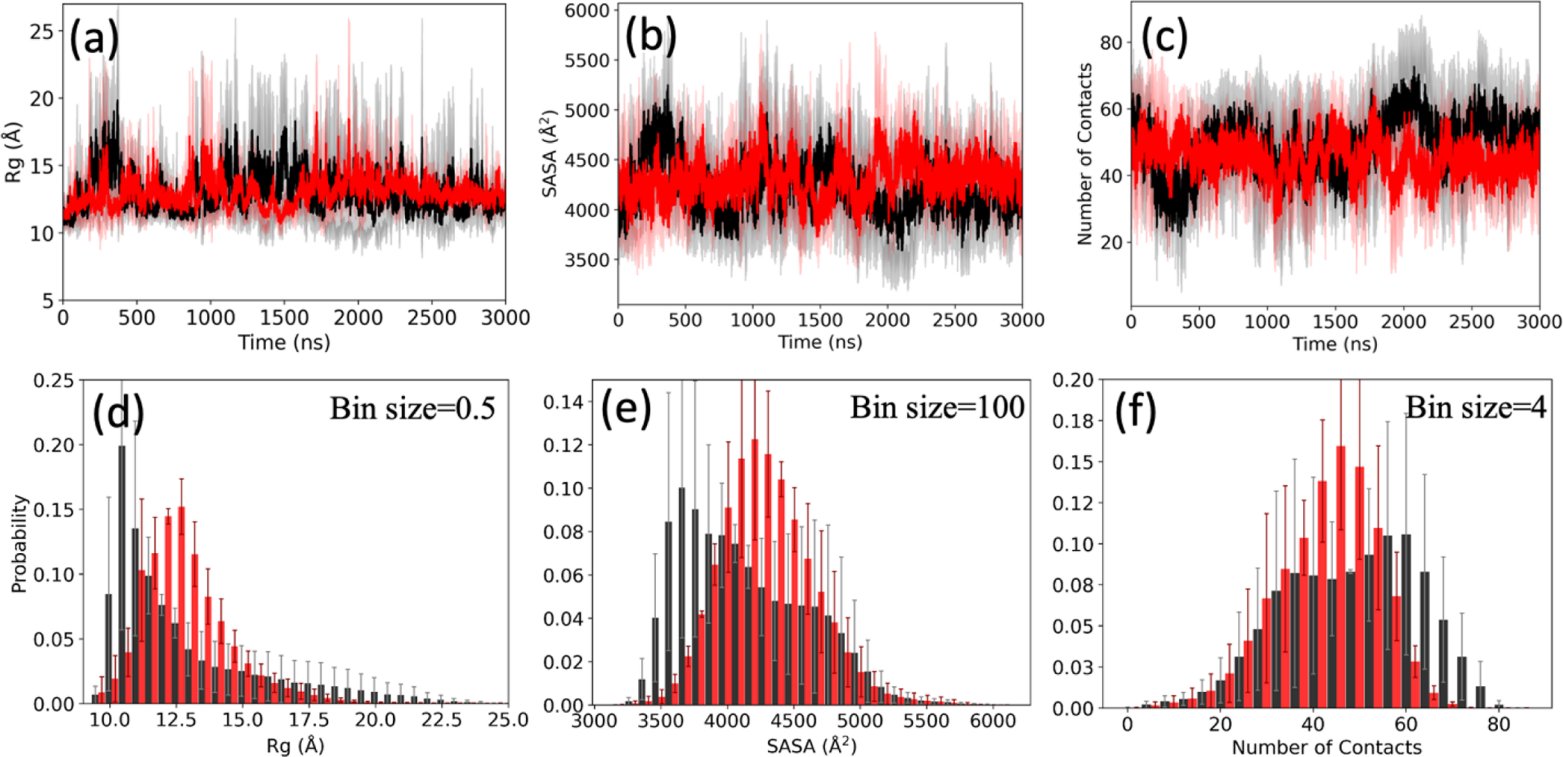
(a) Radius of gyration (*R*_g_), (b) solvent-accessible surface area (SASA), and (c) number of contacts (*n*_C_) of the Aβ_1–42_ monomer in the absence (black) and presence (red) of FI10 as a function of time. The difference from the corresponding value at time *t =* 0 is plotted. The plotted values are averages over three independent trajectories, and the shaded regions mark the standard deviation of the averages. In (d–f), the corresponding normalized averaged distributions of the sampled data collected over the final 2.0 μs of each trajectory.

These differences between simulations in the absence and presence of FI10 seem to be associated with a lower flexibility of the C-terminal residues 30–42, leading to increased values for the residue-wise root-mean-square fluctuations shown in Figure 3a that are correlated with an increased binding propensity of FI10 to C-terminal Aβ_1–42_ residues in Figure 3b. Note, however, that this correlation is much weaker for residues 15–20, which show similar binding propensities. On the other hand, the time series of residue-wise secondary structure for all six trajectories in Figure 4 seems to indicate a higher strandness in the C-terminus when interacting with FI10 (Figure 4b) compared to what is seen in the control (Figure 4a); however, this signal is weak.

**Figure 3:**
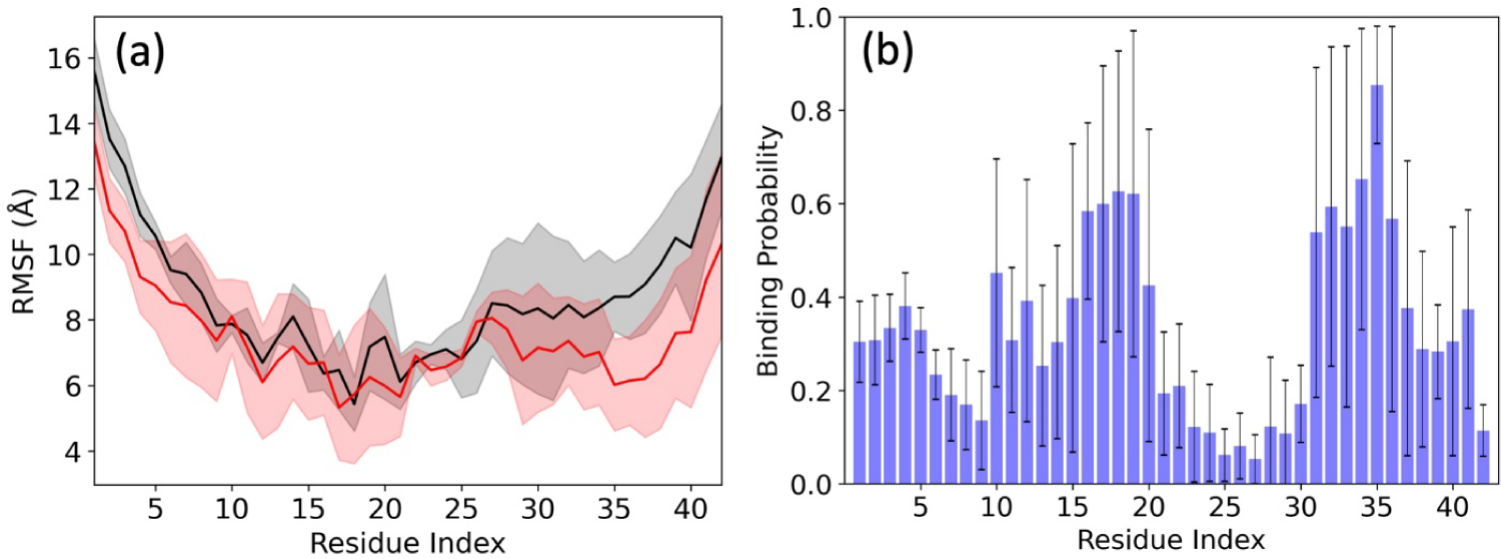
(a) Residue-wise mean square fluctuation (RMSF; in Å) obtained from Aβ_1–42_ monomer simulations in the absence (black) and presence (red) of the FI10 segment. The binding probability of the FI10 segment to the Aβ_1–42_ monomer residues is shown in (b). Data are averaged over the final two μs of each trajectory. and shaded regions in (a) mark the standard deviations of the averages.

**Figure 4:**
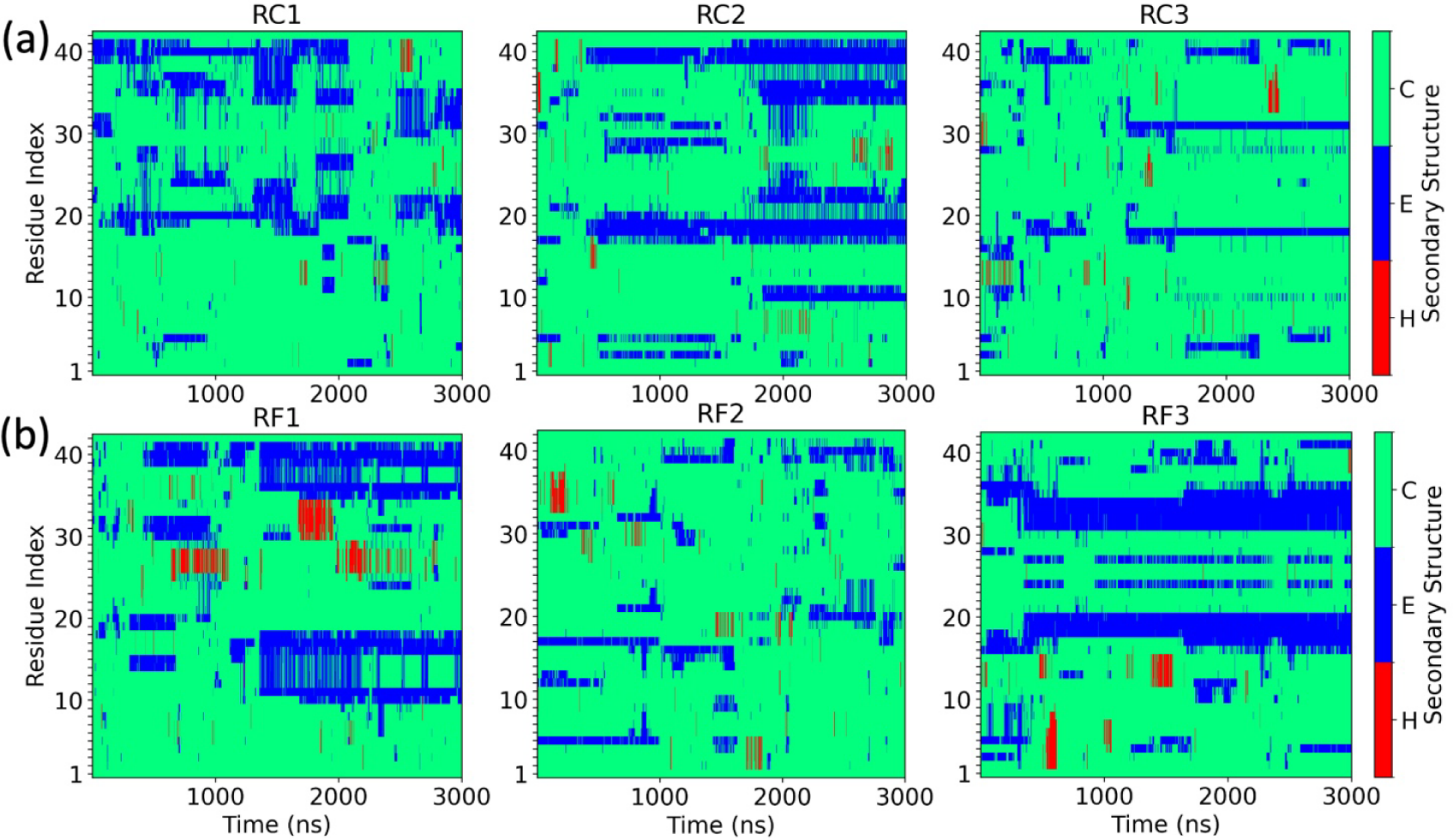
Time series of the residue-wise secondary structure (helix (H), strand (E), and coil (C)) for (a) the three trajectories where FI10 is absent and (b) the three trajectories where FI10 can interact with the Aβ_1–42_ monomer.

In order to get a deeper understanding of the effect of FI10 on the ensemble of Aβ_1–42_ monomers, we have clustered the configurations collected over the last 2 μs in the six trajectories (three for the control and three with FI10 present) according to the procedure described in the Methods section. Considering only clusters containing more than 5% of the configurations in a trajectory, we are left, in the control simulations, with two clusters in trajectory 1, four in trajectory 2, and two in trajectory 3. The frequencies of these clusters are listed in Table 2. Centroids of these eight clusters, comprising together around 53% of the 120000 snapshots in all three trajectories, are shown in Figure 5.

**Table 2:** Frequency of the Eight Most Populous Clusters Found in the Control Simulations, and the Corresponding 14 Clusters Found in the Simulations Where FI10 Interacts with Aβ_1–42_ Monomer[Table-fn tbl2fn1] ^,^ [Table-fn tbl2fn2]

cluster (control)	frequency (%)	cluster (with FI10)	frequency (%)
**Replica 1:** 342 clusters		**Replica 1**: 112 clusters	
RC1_C1	6	RF1_C1	22
RC1_C2	6	RF1_C2	17
**Replica 2:** 72 clusters		RF1_C3	14
RC2_C1	42	RF1_C4	10
RC2_C2	26	RF1_C5	6
RC2_C3	11	RF1_C6	5
RC2_C4	7	**Replica 2**: 385 clusters	
**Replica 3:** 380 clusters		RF2_C1	21
RC3_C1	52	RF2_C2	9
RC3_C2	8	RF2_C3	6
**cumulative frequency:**	**53**	**Replica 3:** 101 clusters	
**Top Two frequency:**	**44**	RF3_C1	29
		RF3_C2	22
		RF3_C3	10
		RF3_C4	7
		RF3_C5	5
		**cumulative frequency:**	**61**
		**Top Two frequency:**	**33**

a Shown are, for both cases, the total number of clusters and the cumulative frequency of the listed most populous clusters.

b Data are taken over the final 2.00 μs of each trajectory. Frequencies are rounded to the closest integer.

**Figure 5:**
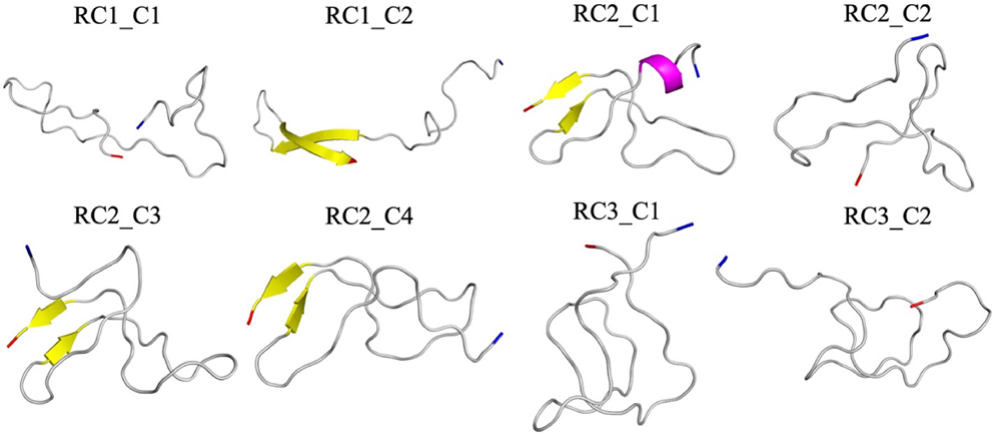
Ribbon representations of the centroid conformations in the eight most populated clusters of configurations sampled over the final 2 μs in the three control trajectories (where FI10 is absent). The N- and C-termini are colored blue and red, respectively.

In a similar way, we find in the three trajectories following simulations of Aβ_1–42_ monomers interacting with FI10, six clusters containing more than 5% of the configurations in trajectory 1, three in trajectory 2, and five clusters in trajectory 3, with the frequencies of these 14 clusters, comprising together about 61% of the 120000 snapshots in all three trajectories, again listed in Table 2, and their centroids shown in Figure 6.

**Figure 6:**
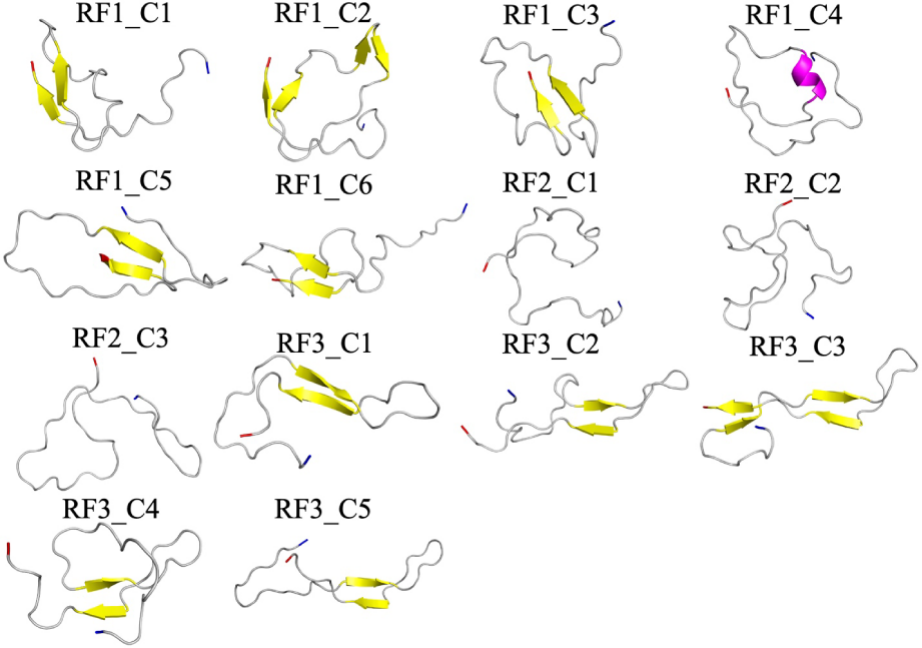
Ribbon representations of the centroid conformations in the 14 most populated clusters of configurations sampled over the final 2 μs in the three trajectories of simulations with FI10 present. The N- and C-termini are colored blue and red, respectively.

We notice that in both sets of simulations, the clusters differed between the three trajectories in each set (which started from different initial configurations). This suggests that an exhaustive sampling of Aβ_1–42_ monomers may require even more than 3 μs of our trajectories. However, we also observe that each set shares common characteristics, which leads us to believe that the main features of the conformational landscape were sampled adequately. For instance, in the presence of the FI10 viral fragment, the monomer conformations cluster more tightly into 199 clusters (containing 61% of all conformations) than in the control, where we find 265 clusters containing 53% of all conformations. Visual inspection of the cluster centroids suggests that the eight clusters from the control simulations can be characterized by the presence of certain transient secondary structure elements: β-strands containing three or more residues in segment 16KLVFFAEDV24 (strand M1), 30AIIGLMV36 (strand C1), or 37GGVVIA42 (strand C2). This is confirmed by the corresponding plots of the strandness as a function of residue number in Figure S2, where these segments appear as clearly identifiable regions. Note that strand M1 corresponds to the hydrophobic segment M1 of Figure 1, and strands C1 and C2 together form the hydrophobic C-terminal segment C of Figure 1. In six of the eight clusters (RC1_C1, RC1_C2, RC2_C1, RC2_C2, RC2_C3, RC2_C4), strand M1 appears in conjunction with C1 or C2; see the frequencies and lifetimes of the three strand segments of the various clusters listed in Table 3, which also shows the respective averages taken over all control simulations. The correspondence of frequencies and similarities in lifetimes of M1 with C1 and C2, and the joint probabilities of M1∩C1 and M1∩C2 in Table S1 indicate that the presence of strand M1 is correlated with that of strand C2 and, to a lesser degree, C1, suggesting the formation of a β-sheet between M1 and C2, which sometimes may also include C1. We remark that this sheet resembles the antiparallel β-sheet between the central hydrophobic core and the segment 29–36 seen in an NMR study^50^ and earlier computational studies.^51,52^

**Table 3:** Frequency and Lifetimes of the Strandness of the Segments 10YEVHH14 (N1), 16KLVFFAEDV24 (M1), 30­(AIIGLMV)­36 (C1), and 37GGVVIA42 (C2) in All Considered Clusters and Averaged over All Configurations Sampled in the Final 2 μs of Each Trajectory^a^ ^b^

	segments							
	N1		M1		C1		C2	
	frequency (%)	lifetime (ps)	frequency (%)	lifetime (ps)	frequency (%)	lifetime (ps)	frequency (%)	lifetime (ps)
Control	0 (0)	104 (139)	44 (36)	592 (169)	29 (22)	541 (144)	31 (28)	539 (66)
with FI10	14 (17)	347 (402)	55 (44)	1214 (1403)	35 (56)	1188 (1671)	31 (29)	553 (212)
Clusters								
RC1_C1	0	50	37	266	14	151	36	267
RC1_C2	1	50	88	231	36	157	86	228
RC2_C1	0	0	85	527	81	605	80	427
RC2_C2	0	0	66	747	0	0	11	470
RC2_C3	0	0	88	415	74	308	86	411
RC2_C4	0	0	86	298	44	208	84	305
RC3_C1	0	0	1	123	1	155	8	384
RC3_C2	0	50	2	63	1	75	0	0
RF1_C1	52	235	62	406	8	671	74	389
RF1_C2	15	164	66	308	19	357	69	340
RF1_C3	65	337	76	996	0	0	80	902
RF1_C4	33	165	55	226	0	0	71	236
RF1_C5	3	142	0	0	0	0	73	707
RF1_C6	23	128	57	182	0	0	63	177
RF2_C1	0	0	23	154	0	100	23	154
RF2_C2	0	0	0	0	0	0	0	0
RF2_C3	0	0	49	458	0	0	38	388
RF3_C1	6	307	100	1546	100	1555	9	330
RF3_C2	0	0	99	2276	100	2786	1	80
RF3_C3	38	380	100	346	100	346	40	363
RF3_C4	0	0	100	3408	100	3882	0	0
RF3_C5	7	157	100	562	100	562	21	258

a Values are rounded to the nearest integer.

b Standard deviations are given within the brackets.

Similarly, in the simulations where FI10 is present, one can also characterize the clusters by the presence of transient secondary structure elements. However, besides the β-strands M1, C1, and C2, we also find an N-terminal β-strand, N1, centered around residue 10YEVHH14, and an α-helix made of residues A30–L34. Note, however, that averaged over the three trajectories, less than 3% of the configurations contain transient helices, while about 15%–20% of conformations have the strand-like segments N1, M1, C1, or C2. The appearance of the N-terminal strand segment N1 is also interesting, as mutations in the N-terminus can be both protective or pathogenic, but their role in aggregation has been poorly studied due to their highly disordered structure in Aβ_1–42_ fibrils;^49^ see also ref 49 for an extensive review. Similar to the control simulations, M1 seems to form, in six clusters (RF2_C1, RF2_C3, RF3_C1, RF3_C2, RF3_C4, RF3_C5), a β-sheet with either C1 or C2; see the frequencies and lifetimes of the four strand segments in Table 3, and the corresponding joint probabilities M1∩C1, N1∩C1, M1∩C2, and N1∩C2 in Table S1. The values in the two tables indicate that, for six other clusters (RF1_C1, RF1_C2, RF1_C3, RF1_C4, RF1_C6, RF3_C3), the formation of a β-sheet occurs between N1 and C1 or C2, in addition to a sheet between M1 and C1 or C2. Note that the corresponding plots of the strandness as a function of residue number for the various clusters in Figure S3 indicate that the presence of the viral fragment FI10 seems to shift the preference of residues to be strand-like toward the N-terminus of the segment M1 (residues 16–24), so that sometimes N1 and M1 merge. This corresponds to a broad region of residues 10–20 to which FI10 has high binding affinity (see Figure 3b). At the same time, FI10 also seems to increase the strandness of the C1 segment, which again corresponds to a region of high binding affinity. Lifetimes of the M1 and C1 strands are almost double those in the control (see Table 3) while that of the C2 strand does not change. However, binding of M1 to C1 is often associated with that of N1 to C2, and both have comparable lifetimes. Hence, the presence of FI10 seems to lengthen the strands that interact with each other.

We remark that several studies have claimed that the central hydrophobic core, composed of the M1 segment, has a strong preference for forming β- strands and is highly amyloidogenic,^53–55^ a fluorescence study has shown that the C2 segment, composed of residues 37–42, exhibits a strong fibrillation effect by stabilizing β-hairpin structures.^56^ Several studies have shown that this β-hairpin structure plays a vital role in oligomer formation, and the inhibition of this β-hairpin structure formation has been proposed as a viable strategy to prevent toxic oligomer formation.^57,58^ Hence, while differences in simulation protocols, secondary structure determination methods, and the use of different starting structures and force fields, the frequencies of strands M1 and C1 in our study being higher than in an earlier computational study (where these strands were observed with frequencies of ∼6–8% and ∼8–14%, respectively),^59^ the increase in both the frequency and lifetime of β-strands in the presence of FI10 still supports our hypothesis that the viral protein fragment facilitates Aβ_1–42_ aggregation.

What keeps the strands connected and stabilized? In Table 4 we list the average number of hydrophobic contacts between the segments when in strand-like conformation and compare it with the corresponding numbers for arbitrary residues. On average, about 30 (8) such contacts are found over the whole trajectory in the control simulations. The maximum possible number of contacts is 276 (24 × 23/2), i.e., the density of hydrophobic contacts is, on average, 0.11. On the other hand, if M1 and C1 are in a strand conformation, there are about ten (out of 42 possible) hydrophobic contacts between the two strands, corresponding to a density of 10/ 42, i.e., 0.24. Hence, hydrophobic contacts between residues in these two segments are found 2.2 times more frequently than on average. If M1 and C2 are in a strand conformation, one finds, on average, 16 of 36 possible hydrophobic contacts between the two segments, leading to a density of around 0.44, i.e., such contacts are found 4 times more frequently than on average. In the simulations where FI10 interacts with the Aβ_1–42_ monomer, we observe, on average, 27 (5) contacts between the 24 hydrophobic residues; i.e., the density of such contacts is similar to those in the control simulations. However, we now find almost double the number (≈19) of contacts between M1 and C1, and about half the number (≈9) of contacts between M1 and C2. Added to these numbers is one additional hydrophobic contact between the segments N1 and C1 and three contacts between segments N1 and C2. This increase in the number of hydrophobic contacts and the relative shift between the segments agrees with our previous observation that the presence of FI10 lengthens the strands that interact with each other.

**Table 4:** Number of Hydrophobic Contacts between Residues in the Aβ_1–42_ Monomer for the Full Chain and between Segments N1, M1, C1, and C2 When in a Strand Conformation^a^ ^b^

	average number of hydrophobic contacts				
simulation	full chain	N1–C1	N1–C2	M1–C1	M1–C2
Control	30 (8)	0.2 (4)	0 (0)	10 (3)	16 (1)
FI10	27 (5)	0.9 (3)	3 (4)	19 (3)	9 (4)

a Data are averaged over all configurations sampled in the final 2.00 μs of each trajectory.

b Standard deviations are given in brackets.

We note that we did not observe hydrogen bonds between segments N1 or M1 and C1 and C2 in our simulations, independent of the presence or absence of the viral protein fragment FI10. This observation indicates that the formation of the strands is not driven by forming hydrogen bonds, as one might expect, and is consistent with the transient character of the strands in our simulations. However, the transient β-sheets and strands seen in our monomer simulations may also be stabilized by salt bridges between charged residues. Once formed, these salt bridges would restrict the conformational space of the segments N1, M1, C1, and C2, thereby encouraging the formation of the observed secondary structure elements, and this process may be modulated by the presence of the viral spike protein fragment FI10. We have therefore also looked into the effect of FI10 on salt bridge formation and the correlation between the observed salt bridges and the transient β-sheets and strands seen in our monomer simulations. When restricting ourselves to salt bridges that appear with in more than 5% of snapshots in at least one trajectory, we find three such salt bridges: E11-H13, E22-K28, and D23-K28. The average frequency and lifetimes of these salt bridges are listed in Table 5.

**Table 5:** Average Frequency of the Appearance of Salt Bridges and Lifetime of Salt Bridges^a^ ^,^ ^b^ ^,^ ^c^

	E11-H13		E22-K28		D23-K28	
	control	FI10	control	FI10	control	FI10
Frequency (%)						
Full chain	4 (3)	16 (12)	23 (25)	6 (5)	4 (4)	29 (38)
N1	0 (0)	30 (15)	29 (26)	2 (1)	1 (2)	24 (28)
M1	5 (3)	16 (12)	10 (12)	3 (3)	2 (2)	47 (32)
C1	7 (3)	8 (6)	8 (9)	1 (3)	3 (3)	68 (17)
C2	5 (2)	24 (11)	4 (13)	7 (4)	0 (0)	16 (23)
N1∩C1	0 (0)	7 (12)	16 (37)	2 (3)	0 (0)	63 (28)
N1∩C2	0 (0)	29 (15)	15 (35)	2 (1)	2 (4)	24 (28)
M1∩C1	6 (3)	8 (4)	8 (10)	1 (3)	3 (3)	70 (13)
M1∩C2	5 (2)	26 (12)	1 (1)	6 (3)	0 (0)	18 (25)
Lifetime (ps)						
Full chain	105 (5)	115 (18)	614 (282)	388 (199)	754 (381)	928 (502)
N1	0 (0)	108 (20)	163 (53)	130 (14)	0 (0)	268 (159)
M1	103 (12)	103 (2)	322 (9)	128 (44)	472 (248)	789 (381)
C1	103 (7)	102 (9)	152 (167)	96 (12)	327 (107)	1062 (267)
C2	93 (21)	105 (10)	193 (40)	224 (49)	495 (251)	349 (94)
N1∩C1	0 (0)	137 (29)	8 (18)	106 (14)	0 (0)	500 (223)
N1∩C2	0 (0)	109 (22)	0 (0)	128 (15)	0 (0)	284 (177)
M1∩C1	96 (8)	103 (3)	157 (165)	94 (6)	312 (118)	1060 (192)
M1∩C2	97 (6)	106 (10)	181 (33)	165 (37)	514 (242)	345 (101)

a Shown are values calculated over all respective trajectories, as well as those restricted to cases where the segments 10YEVHH14 (N1), 16KLVFFAEDV24 (M1), 30­(AIIGLMV)­36 (C1), and 37GGVVIA42 (C2) are in a strand conformation.

b Data are averaged over all configurations sampled in the final 2.00 μs of each trajectory.

c Standard deviations are given in the brackets. Values are rounded to the closest integer.

Our data in Tables 3 and S1 indicate that the four observed strands form transiently even in the control, but the frequency of strands and their pairing is higher in the presence of FI10. On the other hand, when considering the frequencies of the three salt bridges calculated for each system over all three trajectories and listed in Table 5, we see that the salt bridge E22-K28 is more common in the control than in the presence of FI10, while the opposite is true for the salt bridge between E11 and H13, and the one between D23 and K28. The reduced frequency of the salt bridge E22-K28 and its halved lifetime in the presence of FI10 correlate with the binding pattern of the viral protein fragment to the Aβ_1–42_ monomer in Figure 3, i.e., it appears that the binding of FI10 to the monomers interferes with the formation of the salt bridge. We also note that, while the standard deviations in our frequencies are large, even in the control, the existence of this salt bridge seems to be anticorrelated with the strandness in the segments N1, M1, C1, and C2.

On the frequency and lifetime of the D23-K28 salt bridge, these are not only higher in the presence of FI10 than in the control but also larger when the sheets between N1 and C1 or between M1 and C1 are formed, and similarly for the sheets between N1 and C2 and between M1 and C2. These results are consistent with many experimental and theoretical studies showing that the intramolecular D23-K28 salt bridge facilitates the oligomer stability and fibrillization process of Aβ_1–40_ and Aβ_1–42_ peptides.^59–63^ While the salt bridge is not found in all resolved Aβ-fibril models, one could speculate that the enhanced lifetime and frequency of the D23-K28 salt bridge are caused by the presence of FI10, which facilitates the aggregation of Aβ_1–42_ monomers by encouraging formation or stabilizing this salt bridge. Note, however, that the probability of finding the D23-K28 salt bridge is higher under the condition that both N1 and C1 (or M1 and C1) are formed than under the condition that N1 or M1 are formed independently of whether C1 forms a strand. This indicates that the presence of this salt bridge depends on the formation of a sheet between the segments N1 and C1, or between segments M1 and C1, i.e., the D23-K28 salt bridge is formed after the sheet between segments M1 and C1, not causing it. Note also that the sheet between segments M1 and C1 is found in control simulations with a probability of 18% and a lifetime of 750 ps, while the corresponding frequency in the presence of FI10 is 34% with a lifetime of 1000 ps. In the presence of the D23-K28 salt bridge, frequency and lifetime in the control simulations increase to 29% and 750 ps, while in the FI10 simulations, the lifetime stays unchanged and the frequency increases to about 82%, see Table S2. Hence, it appears that the sheet formation is not caused by the D23-K28 salt bridge but rather by direct interaction with the viral fragment or induced hydrophobic contacts.

A similar picture is seen for the salt bridge E11-H13, which appears in the control simulations with about 4%, but in the presence of FI10, it increases to around 16% (see Table 5). In the presence of FI10, this frequency further increases to about 30% if the strand N1 is formed, a value that is similar to the one observed in the presence of sheets between segments N1 and C2 or M1 and C2. Note that little differences are seen in the lifetimes of the salt bridge, neither changing with the presence of FI10 nor depending on the presence of strands. Considering the opposite relationship, i.e., the frequency of strand formation in the segments as a function of the presence of the E11-H13 salt bridge (Table S2), we see that even in the control, the presence of the salt bridge increases the strandness of the segments M1, leading to a higher frequency for the sheets between M1 and C1 or M1 and C2. Correspondingly, in the presence of FI10, the existence of the E11-H13 salt bridge implies a higher frequency for the sheets between segments N1 and C2, and M1 and C2. Hence, the formation of the N1 strand is likely a result of the binding of FI10 to residues Y10 to F20 (segment N1 and parts of M1), which also increases the probability of forming the E11-H13 salt bridge. Once this salt bridge is formed, it leads to the observed pattern of residues 10–20 interacting with residues 30–40. Hence, unlike the D23-K28 salt bridge, we find that the presence of FI10 increases the frequency of the E11-H13 salt bridge, which in turn eases interaction between the N-terminal and C-terminal segments.

### Fibril Simulations

Aβ_1–42_ amyloids form by the association of monomers, but the propensity of amyloids depends not only on the ensemble of monomers (containing more or less aggregation-prone conformations) but also on the stability of the final product of these associations, the experimentally observed fibrils. Hence, viral protein fragments can enhance Aβ-amyloid formation both by shifting the monomer ensemble toward more aggregation-prone conformations and by altering the stability of the fibrils. In previous work, we have shown such stabilization by the FI10 fragment for αS fibrils and found that the effect is differential, i.e., it depends on the specific fibril structure.^13^ Aβ-fibrils are characterized by a large polymorphism, with the structural differences correlating with the severity of disease symptoms.^20,64^ Hence, differential stabilization of Aβ_1–42_ fibrils could potentially hasten the outbreak and/or pathogenesis of Alzheimer’s disease. For this reason, we also considered in this work the effect of FI10 fragments on two distinct fibril models. The first one, having the PDB ID 7Q4B,^65^ is derived from the brains of deceased Alzheimer’s disease patients, while the second one (with PDB ID 5KK3 ^66^) is a synthetic fibril, i.e., the aggregation happened *in vitro* and not in the brain environment. The choice of the two models therefore allows us to probe whether FI10 alters the stability of Aβ_1–42_ fibrils and whether this effect depends on the fibril structure and, therefore, may modulate the pathogenesis of Alzheimer’s disease. Cartoons of the two fibril models are shown in Figure 1e,f.

Patient-derived fibrils of type 1, the ones considered in this study, are present in the brains of sporadic Alzheimer’s disease patients, while the type 2 fibrils (not discussed in this study) are found in the brains of familial Alzheimer’s disease patients.^65^ The fibril is composed of two S-shaped protofilaments, each containing five β-strands per chain. The protofibrils bind through hydrophobic interactions involving the side chains of L34, V36, V39, and I41 residues of the S-shaped domain, as well as Y10, V12, Q15, and L17 of the N-terminal arm.^65^ The synthetic fibril model considered in this study is also composed of two S-shaped protofibrils, each containing four β-strands per chain between residues 16 and 42.^66^ Some hydrophobic interactions, including F19–I32, F19-A30, F20-A24, V24-G29, G29-I41, I31–V36, and G33-V36, play a vital role in determining the fold of the monomer structure in the synthetic fibril. Intermolecular interactions L17-M35 and Q15-M35 have been reported as important interactions between the two protofibrils.^66^ We note that both fibril models do not contain the D23-K28 salt bridge, whose frequency was increased in the presence of FI10 in monomer simulations. Any difference in the way FI10 alters the stability of the two models will therefore not depend on the effect that FI10 has on this salt bridge in the monomer simulations.

We start our investigation by considering, for each system, the root-mean-square deviation (RMSD) to the respective fibril conformation as a function of time. This quantity is calculated for backbone atoms only, ignoring flexible N-terminal residues (the first eight residues of the patient-derived fibril and the first ten residues of the synthetic fibril), and in Figure 7a,b, we show for each system its average over three trajectories. This quantity describes the overall change in the fibril structure along the trajectories, while the change in the structure of individual chains is described by the chain RMSD, which is the average over the RMSD calculated separately for each chain. We show the chain RMSD in the insets. For both fibril models, we observe that the presence of FI10 leads to a slightly higher chain RMSD, i.e., a destabilization of the chain conformations. However, the effect of FI10 on the overall RMSD depends strongly on the fibril model. Little effect is seen for the synthetic fibril (5KK3), while the patient-derived fibril (7Q4B) is destabilized in the presence of FI10. A consequence of these changes is seen in the solvent-accessible surface area (SASA), also shown in Table 6, which, in the patient-derived fibril, shows only marginal changes with time and is not affected by the presence of FI10. However, in the synthetic fibril, the SASA is approximately 264 (5) nm^2^ about 23 nm^2^ larger in the presence of FI10 than in the control (241 (13) nm^2^), with 13 nm^2^ resulting from the addition of hydrophobic surface area. We expect that the differences in the overall RMSD and the SASA indicate different rearrangements of the Aβ_1–42_ in the two fibril geometries over the course of the trajectories caused by the presence of FI10 peptides. As both models are built out of two protofibrils, each made of five layers, changes in the arrangement of the chains can come from loosening or tightening of the layers in each protofibril or from loosening or tightening of the two protofibrils. The first effect can be quantified by the change in the number of stacking contacts between layers, while the second effect is described by the change in packing contacts between the protofibrils.

**Figure 7:**
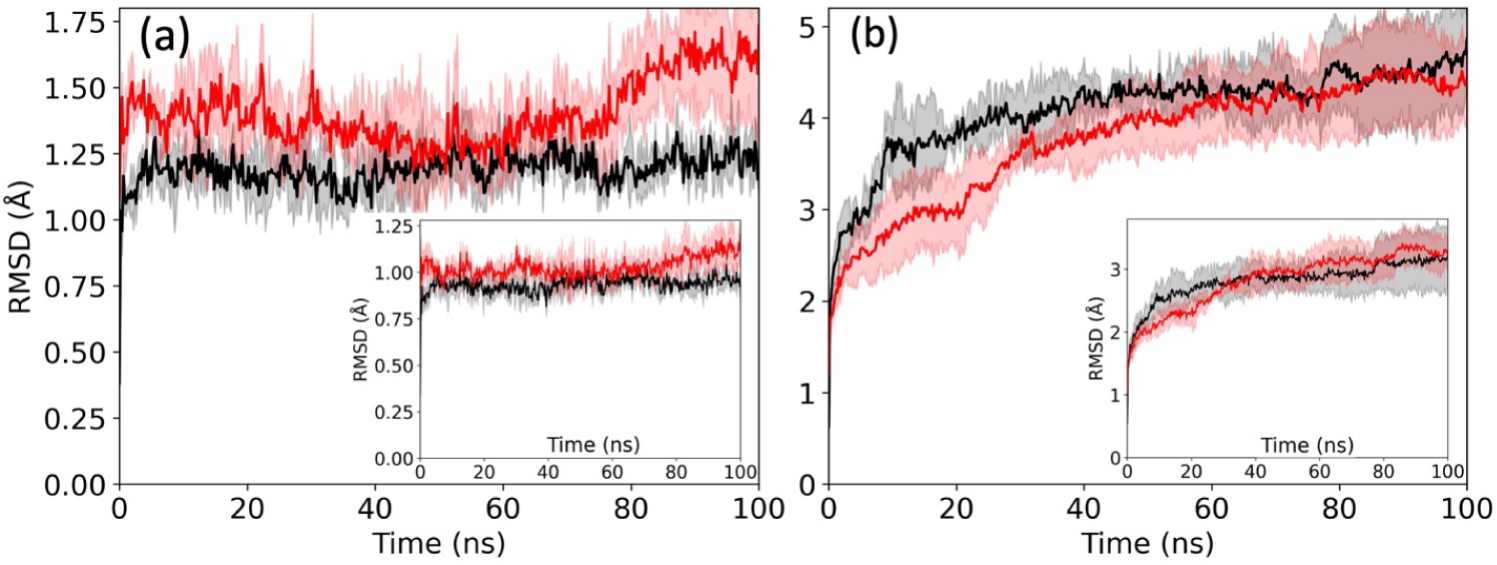
Root-mean-square deviation (RMSD) of the fibril conformations for the patient-derived fibril 7Q4B (a) and the synthetic fibril 5KK3 (b) relative to the respective fibril model (the start configuration) as a function of time. Values for the control are drawn in black, and those from simulations with FI10 present in red. The insets show the chain RMSD, i.e., where the RMSD is calculated separately for each chain and averaged over all ten chains of the system.

**Table 6:** Average Number of Total Contacts, Intrachain Contacts, Staggering Contacts, and Packing Contacts, and Solvent-Accessible Surface Area (SASA), Calculated over the Last 50 ns of All Three Trajectories for Each System^a^ ^,^ ^b^ ^,^ ^c^

	patient-derived fibril (7Q4B)				synthetic fibril (5KK3)			
	control		FI10		control		FI10	
	initial	last 50 ns	initial	last 50 ns	initial	last 50 ns	initial	last 50 ns
Intrachain contacts	16.5 (4)	16.9 (2)	15 (1)	17.0 (3)	13.9 (5)	14 (1)	13.2 (7)	12 (1)
Stacking contacts	170 (2)	169 (1)	168 (2)	166 (1)	148 (6)	139 (7)	143 (2)	142 (5)
Native Stacking Contacts	158 (1)	146 (1)	150 (0)	140 (1)	129 (4)	100 (7)	122 (3)	98 (8)
Packing contacts	99 (6)	120(9)	89 (5)	119 (3)	108 (5)	178 (17)	106 (9)	140 (23)
Native Packing Contacts	86 (7)	71(3)	69 (3)	55 (3)	87 (10)	47 (3)	80 (5)	46 (12)
Long-living Packing Contacts	99 (6)	110 (9)	89 (5)	98 (6)	108 (5)	108(18)	106(9)	98 (12)
Packing Distance (Å)	11.7 (2)	11.3 (1)	12.0 (1)	11.5 (3)	9.3 (2)	9.6 (7)	9.8 (2)	9.2 (4)
SASA (nm^2^)	203 (4)	205 (2)	210 (1)	212 (2)	284 (7)	241 (13)	275(4)	264 (5)
hydrophobic SASA (nm^2^)	93 (2)	94 (1)	100 (0)	98 (1)	144 (3)	120 (8)	142 (2)	133 (3)
hydrophilic SASA (nm^2^)	110 (2)	111 (2)	110 (1)	114 (2)	140 (4)	121 (5)	133 (2)	131 (2)

a The initial values (measured at 0.2 ns to account for steric clashes observed at *t* = 0) for each quantity are also given.

b Note that we do not consider contacts involving the N-terminal residues, as the N-terminal segment is flexible (see main text for details).

c Native contacts are those also seen in the respective fibril models and long-living packing contacts defined in the main text.

In Figure 8a,b, we show the time evolution of the number of staggering contacts (averaged over all three trajectories). Note that we do not consider contacts involving the flexible N-terminal segment added by us, which is missing in the fibril models. For easier comparison, we have normalized the plots such that they equal unity at 0.2 ns, a value chosen to account for steric clashes at *t* = 0 ns. In both the control and in the presence of FI10, we see little change along the trajectories for the patient-derived fibril and a decrease in the absolute number of about ten contacts in the control for the synthetic fibril. When the values are averaged over the final 50 ns, we find for both fibrils similar differences between the control and FI10 simulations, indicating that conformations in the control simulations are stabilized by about 2–3 staggering contacts more than in the simulations where FI10 is present (Table 6). Note that the contacts seen at the end of the trajectories are not necessarily those present in the respective fibril models. The number of these native staggering contacts decreases in all cases (Figure 8c,d), but stays slightly higher for both fibril models in the presence of FI10, as also reflected in the averages over the last 50 ns displayed in Table 6. Hence, the presence of FI10 seems to stabilize the original binding between the layers in both the patient-derived fibril and the synthetic fibril. However, newly formed contacts only partially compensate for the loss of native staggering contacts, and fewer are formed in the presence of FI10, leading, in both fibril models, to an effective weakening through the presence of FI10 by about 2–3 contacts.

**Figure 8:**
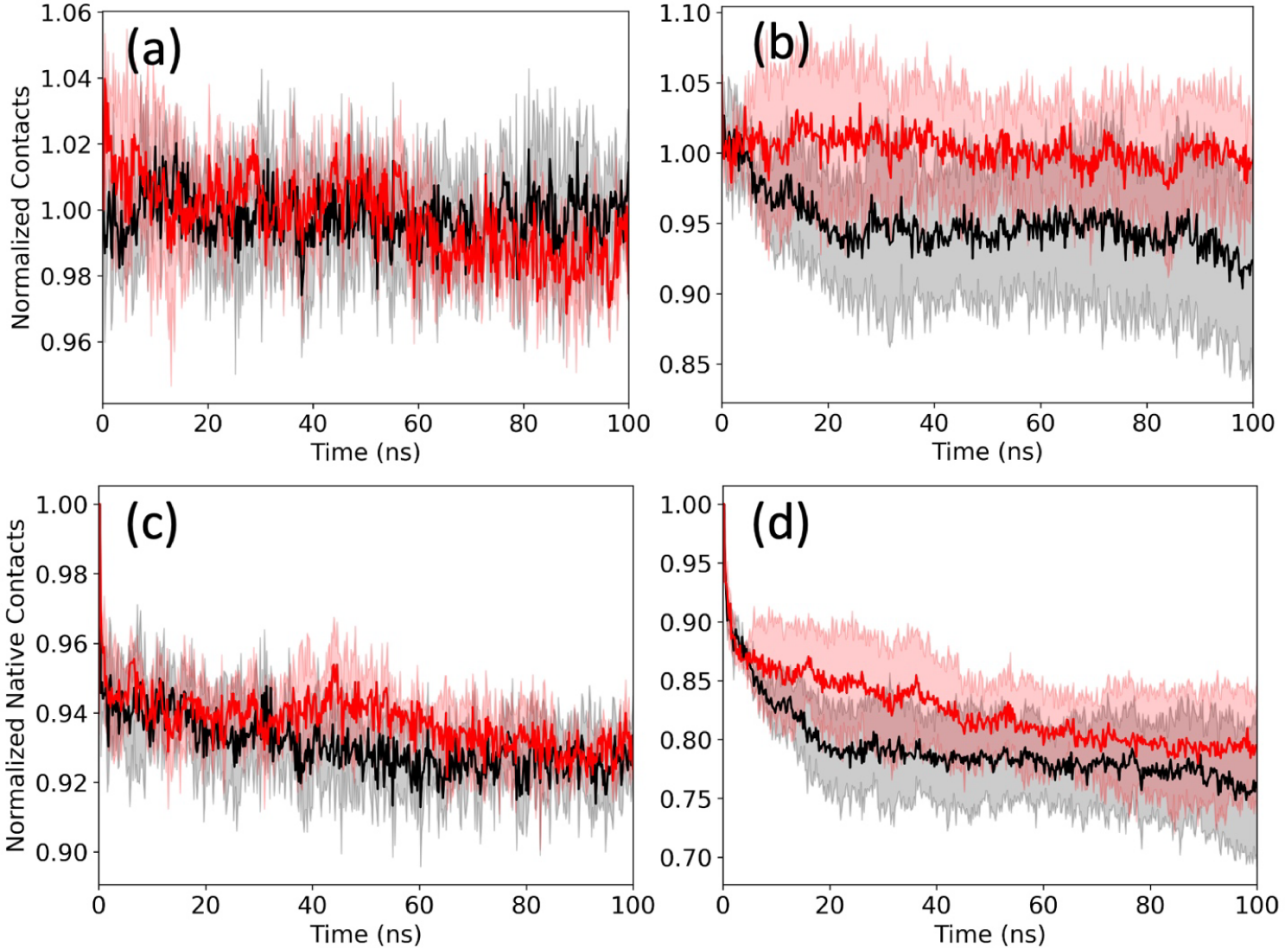
Average number of normalized stacking contacts between residues in neighboring layers of the fibril conformations for the patient-derived fibril 7Q4B (a) and the synthetic fibril 5KK3 (b) as a function of time. Values for the control are drawn in black, and the one from simulations with FI10 present in red. In (c) and (d), we show the corresponding figures for native contacts, i.e., contacts which are seen in the respective fibril models.

The situation is more diverse for the time evolution of normalized packing contacts and native packing contacts, as shown in Figure 9. For the patient-derived fibril, the number of native packing contacts decreases both in the control and in the presence of FI10, with values lower in the presence of FI10. However, the loss of native packing contacts is more than compensated by the formation of new packing contacts. For the control, the absolute number of contacts plateaus quickly, while it continues to grow in the presence of FI10. Final values, averaged over the last 50 ns, are shown in Table 6, indicating that in the presence of FI10, 30 additional contacts are formed, compared to only 21 in the control. For the synthetic fibril, we also observe a decrease in the number of native packing contacts, with a similar loss in both the presence and absence of FI10. Averaged over the last 50 ns, we find 178 (17) contacts in the control simulations but only 140 (23) contacts in the presence of FI10 (see Table 6). We note that we did not see similar differences in the number of intrachain contacts, whose time evolution is shown in Figure S4. Average values for the last 50 ns are also listed in Table 6. Here, the number of intrachain contacts are for the patient-derived 7Q4B fibril, with about 17 contacts, essentially the same in presence and absence of FI10. For the synthetic 5KK3 fibril, the number of intrachain contacts is only marginally smaller in the presence of FI10 (12 (1) versus 14 (1) for the control) confirming our assumption that the presence of FI10 alters the arrangement of the Aβ_1–42_ chains, rather than their structure.

**Figure 9:**
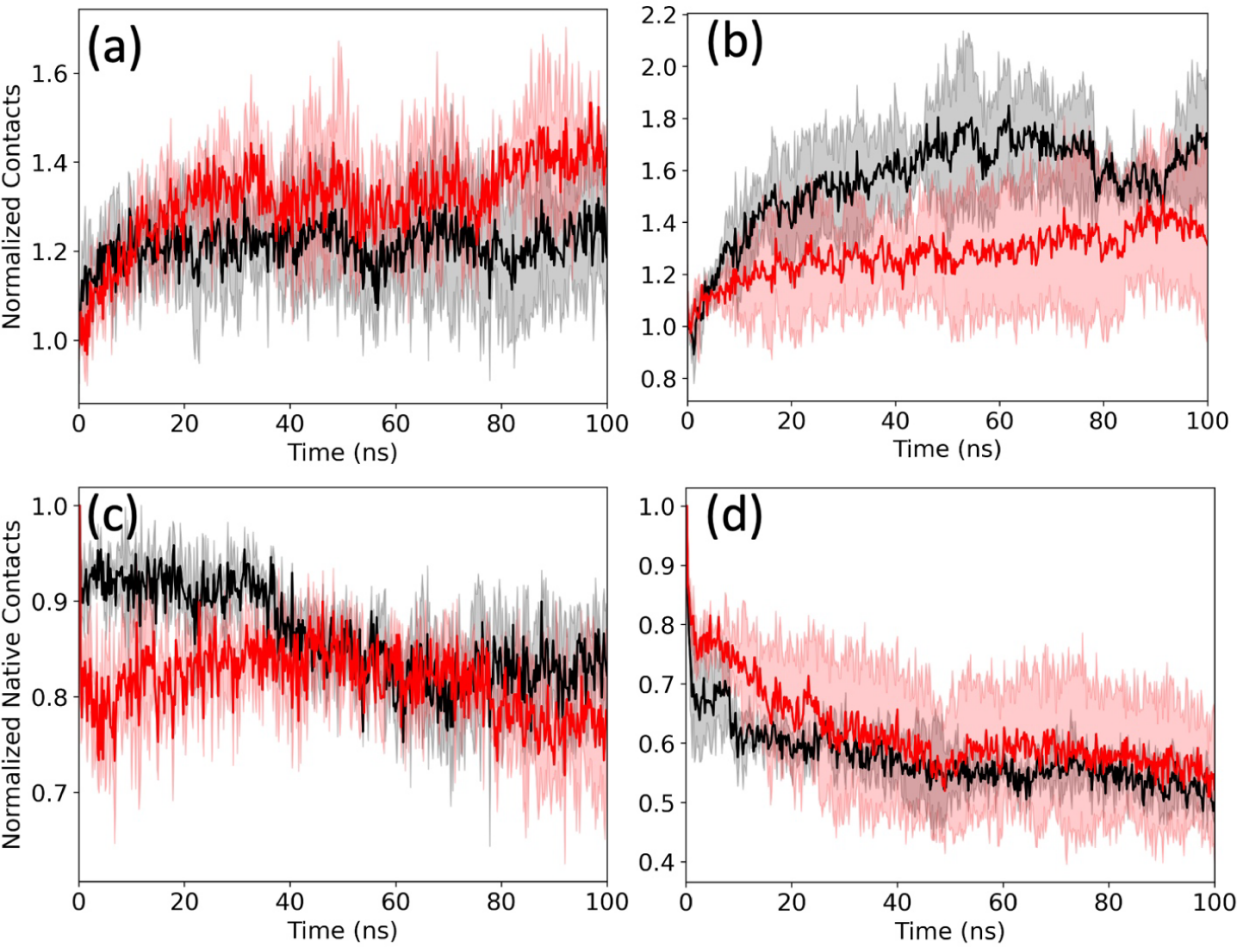
Average number of packing contacts between residues in opposite protofibrils of the fibril conformations for the patient-derived fibril 7Q4B (a) and the synthetic fibril 5KK3 (b) as a function of time. Values for the control are drawn in black, and the one from simulations with FI10 present in red. For better comparison, the values are normalized to unity for the conformation observed at the start. In (c) and (d), we show the corresponding figures for native contacts, i.e., contacts seen in the respective fibril models and the start conformations.

This change in packing contacts is correlated with the packing distance, which is the distance between the two protofibrils. We define packing distance as the distance between the centers of mass of the interfacial residues of two protofibrils. For the patient-derived fibril, we considered Y10, V12, Q15, L17, L34, M35, V39, and I41 as interfacial residues, while the residues H13, H14, Q15, K16, G33, L34, M35, V36, G37, G38, and V39 were considered as interfacial residues of the synthetic fibril. For the patient-derived fibril, this packing distance decreases in the control by about 0.4 and 0.5 Å in the presence of FI10; i.e., the two protofibrils change their relative positions (and therefore the contacts between residues), but their distance to each other is minimally changed and slightly more reduced in the presence of FI10. The stabilizing effect on packing by FI10 is also seen for the synthetic fibril, where the distance decreases in the presence of FI10 but increases in the control. Hence, it appears that FI10 slightly destabilizes the stacking of the Aβ_1–42_ chains in a way that does not depend on the specific fibril type, but has a stronger effect on the packing of protofibrils in a way that depends on the fibril geometry.

For the patient-derived fibril, the main packing contacts in the PDB structures are between V12–V39, L17–V36, L34–V36, V36–L34, V36–L17, and V39–V12, with the residues on different protofibrils and contacts either with residues of the same layer or shifted by one layer. When interacting with FI10, the number of such contacts is reduced from about 44 to 41. However, these contacts survive in both cases throughout the simulations, and the observed changes in the number and type of packing contacts result from the rearrangement of other packing contacts, such as Q15–V39 or F19–V36, with new contacts often forming between residues in different layers in the protofibrils, and their number larger in the presence of FI10 (23 as opposed to 14 in the control), leading to the decrease in packing distance seen in both the control and the presence of FI10. On the other hand, in the synthetic fibril, the dominating contacts are L17–M35, L34–M35, M35–L34, and M35–L17, which decrease from about 30 in both cases at the start to 23 in the control and only 19 in the presence of FI10. Interestingly, the loss of these contacts is mostly for contacts between residues on different layers (about 7), and is for residue pairs located on the same layer smaller than in the control (3 as opposed to 8 in the control).

Note that, as already reported above, the total number of packing contacts increases in both systems. However, most of these newly formed contacts are transient. This can be seen if we measure, instead of the contacts, the number of pairs where the *average distance* between the residues is smaller than the cutoff distance of 4.5 Å used in our definition of a contact. Such pairs correspond to long-living packing contacts, while our previous definition also accounts for short-lived and transient contacts. At the start, we find for the synthetic fibril in the control 108 (5) of such pairs, and in the presence of FI10, 106 (9) pairs, but in the last 50 ns, there are 108 (18) pairs in the control and only 98 (12) in the trajectories where FI10 is present. Hence, the number of long-living contacts decreases, as does the number of native contacts (see above), and the newly formed non-native contacts are therefore short-lived, transitory contacts that do not lead to a decrease in the packing distance, i.e., a stronger interaction between the two protofibrils. This is different in the patient-derived fibril, where the number of long-living packing contacts also increases for the control simulations by about 11 and by about 9 in the presence of FI10. These additional contacts lead to a stronger interaction between the two protofibrils in the presence of FI10, causing a reduction in packing distance and, therefore, stabilizing the fibril.

The observed differences may result from the disparate binding pattern of FI10 and the two fibril geometries (see Figure 10). For the patient-derived fibril, FI10 binds with a free energy of about −90 (8) kJ/mol. Figure 10a shows that the binding of FI10 is preferably to residues 15–23 of the hydrophobic segment M1 (on average 51%) and residues 30–41 of hydrophobic segments C1 and C2 (on average 33% to the C1 segments, with a maximum of 44% at L34, and on average 20% to the C2 segment), with a binding probability of about 70% for the charged residues K16 and E22 or D23. FI10 binds to other parts of the fibril with a much lower affinity of about 7%. On the other hand, the binding distribution is more indiscriminate for the synthetic fibril, with generally higher binding probabilities (on average 35%) but no specific preference for a certain segment (see Figure 10b). When looking into the binding pattern of the FI10 residues, we find that the Aβ_1–42_ chains of the synthetic fibril also bind nonspecifically to FI10 residues (Figure 10f), but at higher binding frequencies than seen for binding to the patient-derived fibril, wherethe binding propensities slightly decrease from the *N*- to the C-terminal of FI10 (Figure 10e). This suggests that, while FI10 binds more tightly to the synthetic fibril, with a free energy of −108 (9) kJ/mol, than to the patient-derived fibril, the peptide ismobile on the synthetic fibril and changes the residues on the chains with which it interacts, while on the patient-derived fibril, it remains more localized. Note that approximating the binding affinity of FI10 to the fibril by the sum of binding energies of the individual residues may overestimate the binding affinity, but this does not change their ranking and therefore does not affect our conclusion. Hence, the lower RMSF and lower flexibility is observed in the synthetic fibril in the presence of FI10 (Figure 10d) while for the patient-derived fibril, the interaction with FI10 leads to increased flexibility of the noninteracting chain segments, especially at the N-terminus (Figure 10c).

**Figure 10:**
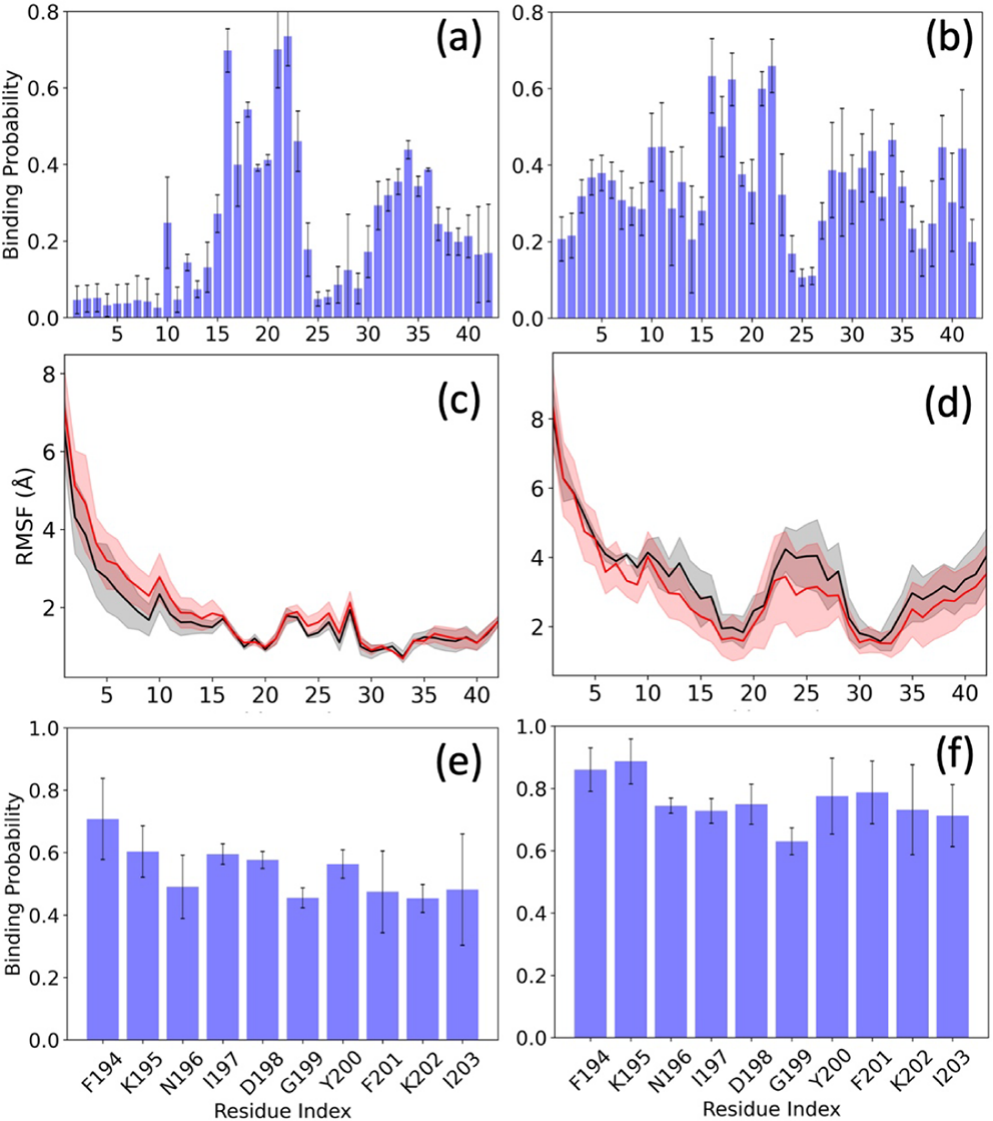
Residue-wise normalized binding probability of the FI10 segment for the Aβ_1–42_ chains is shown in (a) for the patient-derived fibril model 7Q4B and in (b) for the synthetic fibril model 5KK3, while the corresponding residue-wise mean square fluctuation (RMSF; in Å) obtained from Aβ_1–42_ fibril simulations in the absence (black) and presence red) of FI10 is shown in (c) and (d). In (e), we show the complementarity, and in (f), the binding probability of residues in the synthetic fibril model 5KK3 to FI10 residues. Data are averaged over the final 50 ns of each trajectory. Shaded regions or error bars marks the standard deviation of the averages.

## Conclusions

In order to understand the effect of small viral protein fragments on the aggregation of Aβ_1–42_ peptides as a potential factor in the initiation and progression of Alzheimer’s disease, we have performed all-atom molecular dynamics simulations of Aβ_1–42_ monomer and fibril in the presence of the SARS-CoV-2 Spike protein fragment 194FKNIDGYFKI203 (FI10). Our simulations indicate that FI10 shifts the ensemble of monomers to less compact conformations with a larger surface exposed to the solvent, stabilizing especially the C-terminal segment of residues 30 to 42. While even in the absence of FI10 strand-like segments 16KLVFFAEDV24 (strand M1), 30AIIGLMV36 (strand C1), or 37GGVVIA42 (strand C2) form, the presence of FI10 increases their frequency and lifetimes and extends the segment M1 toward the N-terminus or adds a strand 10YEVHH14 (N1). The N- and C-terminal segments are stabilized by hydrophobic contacts between the segments, which are higher in the presence of FI10; further stabilization is provided by salt bridges D23-K28 and E11-H13 forming subsequently. Hence, the presence of FI10 will increase the propensity of Aβ_1–42_ monomers to aggregate. However, this increase is unspecific, as the aggregation-prone strand segments appear not to favor a specific fibril polymorph and are found in many of the experimentally resolved fibril models, see, for instance, the fibril models with PDB IDs 7Q4B, 7Q4M, 5KK3, 8EZD.

As the equilibrium between monomers and amyloids can also be shifted by stabilizing the end product of this process, we have investigated, in complementary simulations, two distinct fibril fragments: the patient-derived 7Q4B and the synthetic 5KK3, as both contain the above-described segments N1, M1, C1, and C2. Analyzing our trajectories, we find no change caused by FI10 on the chain conformations (for instance, in the number of intrastrand contacts) but rather on the relative arrangement of the chains in the fibrils. FI10 may slightly destabilize the stacking of the Aβ_1–42_ chains in a way that does not depend on the specific fibril type, but has a stronger effect on the packing of protofibrils (as seen in the number of packing contacts, packing distance, and the solvent-exposed surface area), which depends on the fibril geometry. Especially, we find that the presence of FI10 leads to a stronger packing of the protofibrils in the patient-derived fibril, while this effect is only marginal for the synthetic fibril. Hence, while FI10 appears to increase the aggregation propensity of the monomers without an obvious preference for a specific outcome, it stabilizes the fully formed fibrils differently depending on their geometry. We relate this differential stabilization to the more localized binding of FI10 to the patient-derived fibril than seen in the synthetic fibril, where FI10 binds diffusively.

Comparing our results for Aβ_1–42_ monomers and fibrils with earlier work, we find a common theme: FI10 and other SARS-CoV-2 protein fragments can indeed increase amyloid formation of human proteins such as αS, SAA, and amylin.^12,13,22,23^ As such small viral protein fragments are likely present *in vivo* following cleavage during infection-caused inflammation, amyloid diseases may be triggered in this way by SARS-CoV-2 and likely also other viral infections. A common feature in all cases seems to be that the viral protein fragments appear to shift the ensemble of monomer conformations toward more aggregation-prone ones. Less clear is the effect on the end product—the fibrils often seen as a hallmark of various diseases. Here, we find that the modulation of stability of the potentially cell-toxic fibrils depends on the geometry of the fibrils. In the case of Aβ_1–42_ it appears that fibril polymorphs are stabilized, which are found in patients with Alzheimer’s disease. However, our study looked only into two of many experimentally resolved fibrils, and more work is needed to establish whether disease-relevant geometries are indeed preferentially stabilized by the viral proteins. Further work is also needed to investigate the effect of FI10 and similar fragments on intermediates such as dimers and other oligomers in the aggregation pathways that would seed fibril formation. This is especially important in the case of Aβ, where oligomers are considered to be more likely disease-causing agents than fibrils.

## Supplementary Material





## Data Availability

The data supporting the results of this study are available in the Supporting Information and are publicly accessible at https://github.com/ouhansmannlab/amyloid_beta

## Notes

Ribbon representations of the three initial configurations of Aβ_1‑42_ monomers and the two fibril models, 7Q4B and 5KK3 bound with FI10 peptides in a 1:1 ratio (Figure S1); average strandness as a function of residues in the eight most populated clusters seen in the control simulations (Figure S2); average strandness as a function of residues in the 14 most populated clusters seen in the simulations where FI10 is present (Figure S3); average number of intrachain contacts between residues in the fibril conformations for the patient-derived fibril 7Q4B and the synthetic fibril 5KK3 as a function of time (Figure S4); joint frequency (in percent) for pairs of the segments N1–C1, N1–C2, M1–C1, and M1–C2 being in a strand conformation (Table S1); frequency of strandness for the segments N1, M1, C1, and C2 as a function of the presence of the three salt bridges E1-H13, E22-K28, and D23-K28 (Table S2) (PDF)
Atomic coordinates of selected Aβ_1–42_ monomers and fibril conformations (ZIP)

## Abbreviations

AD: Alzheimer’s disease
Aβ: Amyloid β
αS: α synuclein
DSSP: Dictionary of secondary structure in proteins
HIV-TAT: Human immunodeficiency virus transactivator of transcription
HSV-1: Herpes simplex virus 1
MD: Molecular dynamics
PD: Parkinson’s disease
PDB: Protein data bank
PFC: Patient-derived fibril control
PFF: Patient-derived fibril with FI10
PME: Particle Mesh Ewald
RC1: Replica 1 Control
RC1_C1: Replica 1 Control-Cluster 1
RC2: Replica 2 Control
RC3: Replica 3 Control
RF1: Replica 1 with FI10
RF1_C1: Replica 1 with FI10-Cluster 1
RF2: Replica 2 with FI10
RF3: Replica 3 with FI10
Rg: Radius of gyration
RMSD: Root mean square deviation
RMSF: Root mean square fluctuation
SAA: Serum amyloid A
SARS-CoV-2: Severe acute respiratory syndrome coronavirus-2
SASA: Solvent accessible surface area
SFC: Synthetic fibril control
SFF: Synthetic fibril with FI10

## References

[1] Eisenberg D., Jucker M. (2012). The Amyloid State of Proteins in Human Diseases. Cell.

[2] Labrie V., Brundin P. (2017). Alpha-Synuclein to the Rescue: Immune
Cell Recruitment
by Alpha-Synuclein during Gastrointestinal Infection. J. Innate Immun.

[3] Eimer W. A., Vijaya Kumar D. K., Navalpur Shanmugam N.
K., Rodriguez A. S., Mitchell T., Washicosky K. J., György B., Breakefield X. O., Tanzi R. E., Moir R. D. (2018). Alzheimer’s
Disease-Associated β-Amyloid Is Rapidly Seeded by Herpesviridae
to Protect against Brain Infection. Neuron.

[4] Kumar D. K. V., Choi S. H., Washicosky K. J., Eimer W. A., Tucker S., Ghofrani J., Lefkowitz A., McColl G., Goldstein L. E., Tanzi R. E., Moir R. D. (2016). Amyloid-β
Peptide Protects
against Microbial Infection in Mouse and Worm Models of Alzheimer’s
Disease. Sci. Transl. Med.

[5] Fulop T., Witkowski J. M., Bourgade K., Khalil A., Zerif E., Larbi A., Hirokawa K., Pawelec G., Bocti C., Lacombe G., Dupuis G., Frost E. H. (2018). Can an
Infection
Hypothesis Explain the Beta Amyloid Hypothesis of Alzheimer’s
Disease?. Front. Aging Neurosci.

[6] Semerdzhiev S. A., Fakhree M. A. A., Segers-Nolten I., Blum C., Claessens M. M. A. E. (2022). Interactions
between SARS-CoV-2 N-Protein and α-Synuclein Accelerate Amyloid
Formation. ACS Chem. Neurosci.

[7] Haas J. G., Lathe R. (2018). Microbes and Alzheimer’s
Disease: New Findings Call for a
Paradigm Change. Trends Neurosci.

[8] Mesias V. S. D., Zhu H., Tang X., Dai X., Liu W., Guo Y., Huang J. (2022). Moderate Binding between
Two SARS-CoV-2 Protein Segments
and α-Synuclein Alters Its Toxic Oligomerization Propensity
Differently. J. Phys. Chem. Lett.

[9] Moir R. D., Lathe R., Tanzi R. E. (2018). The Antimicrobial Protection Hypothesis
of Alzheimer’s Disease. Alzheimers Dement.

[10] Al-Kuraishy H. M., Al-Gareeb A. I., Kaushik A., Kujawska M., Ahmed E. A., Batiha G. E. (2023). SARS-COV-2 Infection and Parkinson’s Disease:
Possible Links and Perspectives. J. Neurosci.
Res.

[11] Makhoul K., Jankovic J. (2021). Parkinson’s Disease after COVID-19. J. Neurol. Sci.

[12] Jana A. K., Lander C. W., Chesney A. D., Hansmann U. H. E. (2022). Effect of an
Amyloidogenic SARS-COV-2 Protein Fragment on α-Synuclein Monomers
and Fibrils. J. Phys. Chem. B.

[13] Chesney A. D., Maiti B., Hansmann U. H. E. (2023). SARS-COV-2 Spike Protein Fragment
Eases Amyloidogenesis of α-Synuclein. J. Chem. Phys.

[14] Nyström S., Hammarström P. (2022). Amyloidogenesis of SARS-CoV-2 Spike Protein. J. Am. Chem. Soc.

[15] Wang L., Davis P. B., Volkow N. D., Berger N. A., Kaelber D. C., Xu R. (2022). Association of COVID-19
with New-Onset Alzheimer’s Disease. J.
Alzheimers Dis.

[16] Domingues R. B., Mendes-Correa M. C., De Moura Leite F. B.
V., Sabino E. C., Salarini D. Z., Claro I., Santos D. W., De Jesus J. G., Ferreira N. E., Romano C. M., Soares C. A. S. (2020). First Case of
SARS-COV-2 Sequencing in Cerebrospinal Fluid of a Patient with Suspected
Demyelinating Disease. J. Neurol.

[17] Meinhardt J., Radke J., Dittmayer C., Franz J., Thomas C., Mothes R., Laue M., Schneider J., Brünink S., Greuel S., Lehmann M., Hassan O., Aschman T., Schumann E., Chua R. L., Conrad C., Eils R., Stenzel W., Windgassen M., Rößler L., Goebel H.-H., Gelderblom H. R., Martin H., Nitsche A., Schulz-Schaeffer W. J., Hakroush S., Winkler M. S., Tampe B., Scheibe F., Körtvélyessy P., Reinhold D., Siegmund B., Kühl A. A., Elezkurtaj S., Horst D., Oesterhelweg L., Tsokos M., Ingold-Heppner B., Stadelmann C., Drosten C., Corman V. M., Radbruch H., Heppner F. L. (2021). Olfactory
Transmucosal SARS-CoV-2 Invasion as a Port of Central Nervous System
Entry in Individuals with COVID-19. Nat. Neurosci.

[18] Song E., Zhang C., Israelow B., Lu-Culligan A., Prado A. V., Skriabine S., Lu P., Weizman O.-E., Liu F., Dai Y., Szigeti-Buck K., Yasumoto Y., Wang G., Castaldi C., Heltke J., Ng E., Wheeler J., Alfajaro M. M., Levavasseur E., Fontes B., Ravindra N. G., Van Dijk D., Mane S., Gunel M., Ring A., Kazmi S. A. J., Zhang K., Wilen C. B., Horvath T. L., Plu I., Haik S., Thomas J.-L., Louvi A., Farhadian S. F., Huttner A., Seilhean D., Renier N., Bilguvar K., Iwasaki A. (2021). Neuroinvasion of SARS-CoV-2 in Human and Mouse Brain. J. Exp. Med.

[19] Ezzat K., Pernemalm M., Pålsson S., Roberts T. C., Järver P., Dondalska A., Bestas B., Sobkowiak M. J., Levänen B., Sköld M., Thompson E. A., Saher O., Kari O. K., Lajunen T., Sverremark Ekström E., Nilsson C., Ishchenko Y., Malm T., Wood M. J. A., Power U. F., Masich S., Lindén A., Sandberg J. K., Lehtiö J., Spetz A.-L., El Andaloussi S. (2019). The Viral
Protein Corona Directs Viral Pathogenesis and Amyloid Aggregation. Nat. Commun.

[20] Hategan A., Bianchet M. A., Steiner J., Karnaukhova E., Masliah E., Fields A., Lee M.-H., Dickens A. M., Haughey N., Dimitriadis E. K., Nath A. (2017). HIV Tat Protein and
Amyloid-β Peptide Form Multifibrillar Structures That Cause
Neurotoxicity. Nat. Struct. Mol. Biol.

[21] Cao S., Song Z., Rong J., Andrikopoulos N., Liang X., Wang Y., Peng G., Ding F., Ke P. C. (2023). Spike Protein Fragments Promote Alzheimer’s
Amyloidogenesis. ACS Appl. Mater. Interfaces.

[22] Jana A. K., Greenwood A. B., Hansmann U. H. E. (2021). Presence of a SARS-CoV-2 Protein
Enhances Amyloid Formation of Serum Amyloid A. J. Phys. Chem. B.

[23] Chesney A. D., Maiti B., Hansmann U. H. E. (2023). Human
Amylin in the Presence of SARS-COV-2
Protein Fragments. ACS Omega.

[24] Yasar F., Ray A. J., Hansmann U. H. E. (2022). Resolution
Exchange with Tunneling
for Enhanced Sampling of Protein Landscapes. Phys. Rev. E.

[25] van
Zundert G. C. P., Rodrigues J. P. G. L.
M., Trellet M., Schmitz C., Kastritis P. L., Karaca E., Melquiond A. S. J., van Dijk M., de Vries S. J., Bonvin A. M. J. J. (2016). The HADDOCK2.2
Web Server: User-Friendly Integrative Modeling of Biomolecular Complexes. J. Mol. Biol.

[26] Honorato R. V., Koukos P. I., Jiménez-García B., Tsaregorodtsev A., Verlato M., Giachetti A., Rosato A., Bonvin A. M. J. J. (2021). Structural Biology in the Clouds:
The WeNMR-EOSC Ecosystem. Front. Mol. Biosci.

[27] Schrödinger, Inc. The PyMOL Molecular Graphics System, Schrödinger, Inc. 2021.

[28] Abraham M.
J., Murtola T., Schulz R., Páll S., Smith J. C., Hess B., Lindahl E. (2015). GROMACS: High Performance
Molecular Simulations through Multi-Level Parallelism from Laptops
to Supercomputers. SoftwareX.

[29] Huang J., Rauscher S., Nawrocki G., Ran T., Feig M., de Groot B. L., Grubmüller H., MacKerell A. D. (2017). CHARMM36m:
An Improved Force Field for Folded and Intrinsically Disordered Proteins. Nat. Methods.

[30] Jorgensen W. L., Chandrasekhar J., Madura J. D., Impey R. W., Klein M. L. (1983). Comparison
of Simple Potential Functions for Simulating Liquid Water. J. Chem. Phys.

[31] Wang W., Hansmann U. H. E. (2020). Stability of
Human Serum Amyloid A Fibrils. J. Phys. Chem.
B.

[32] Pandey P., Nguyen N., Hansmann U. H. E. (2020). d -Retro
Inverso Amylin
and the Stability of Amylin Fibrils. J. Chem.
Theory Comput.

[33] Siwy C. M., Lockhart C., Klimov D. K. (2017). Is the Conformational
Ensemble of
Alzheimer’s Aβ10–40 Peptide Force Field Dependent?. PLoS Comput. Biol.

[34] Samantray S., Yin F., Kav B., Strodel B. (2020). Different Force Fields Give Rise
to Different Amyloid Aggregation Pathways in Molecular Dynamics Simulations. J. Chem. Inf. Model.

[35] Man V. H., He X., Derreumaux P., Ji B., Xie X.-Q., Nguyen P. H., Wang J. (2019). Effects of All-Atom
Molecular Mechanics Force Fields on Amyloid Peptide
Assembly: The Case of Aβ _16–22_ Dimer. J. Chem. Theory Comput.

[36] Bussi G., Donadio D., Parrinello M. (2007). Canonical
Sampling through Velocity
Rescaling. J. Chem. Phys.

[37] Parrinello M., Rahman A. (1981). Polymorphic Transitions
in Single Crystals: A New Molecular
Dynamics Method. J. Appl. Phys.

[38] Miyamoto S., Kollman P. A. (1992). Settle: An Analytical
Version of the SHAKE and RATTLE
Algorithm for Rigid Water Models. J. Comput.
Chem.

[39] Hess B., Bekker H., Berendsen H. J. C., Fraaije J. G. E. M. (1997). LINCS: A Linear
Constraint Solver for Molecular Simulations. J. Comput. Chem.

[40] Essmann U., Perera L., Berkowitz M. L., Darden T., Lee H., Pedersen L. G. (1995). A Smooth Particle
Mesh Ewald Method. J. Chem. Phys.

[41] Humphrey W., Dalke A., Schulten K. (1996). VMD: Visual
Molecular Dynamics. J. Mol. Graphics.

[42] Pettersen E. F., Goddard T. D., Huang C. C., Couch G. S., Greenblatt D. M., Meng E. C., Ferrin T. E. (2004). UCSF Chimera—A Visualization
System for Exploratory Research and Analysis. J. Comput. Chem.

[43] McGibbon R. T., Beauchamp K. A., Harrigan M. P., Klein C., Swails J. M., Hernández C. X., Schwantes C. R., Wang L.-P., Lane T. J., Pande V. S. (2015). MDTraj:
A Modern Open Library for the Analysis of Molecular
Dynamics Trajectories. Biophys. J.

[44] Michaud-Agrawal N., Denning E. J., Woolf T. B., Beckstein O. (2011). MDAnalysis:
A Toolkit for the Analysis of Molecular Dynamics Simulations. J. Comput. Chem.

[45] Kabsch W., Sander C. (1983). Dictionary of Protein
Secondary Structure: Pattern
Recognition of Hydrogen-bonded and Geometrical Features. Biopolymers.

[46] Daura X., Gademann K., Jaun B., Seebach D., van Gunsteren W. F., Mark A. E. (1999). Peptide Folding:
When Simulation Meets Experiment. Angew. Chem.,
Int. Ed.

[47] Bellaiche M. M. J., Best R. B. (2018). Molecular Determinants
of Aβ_42_ Adsorption
to Amyloid Fibril Surfaces. J. Phys. Chem. Lett.

[48] Leguizamon
Herrera V. L., Buell A. K., Willbold D., Barz B. (2022). Interaction
of Therapeutic d -Peptides with Aβ42 Monomers, Thermodynamics,
and Binding Analysis. ACS Chem. Neurosci.

[49] Nasica-Labouze J., Nguyen P. H., Sterpone F., Berthoumieu O., Buchete N.-V., Coté S., De Simone A., Doig A. J., Faller P., Garcia A., Laio A., Li M. S., Melchionna S., Mousseau N., Mu Y., Paravastu A., Pasquali S., Rosenman D. J., Strodel B., Tarus B., Viles J. H., Zhang T., Wang C., Derreumaux P. (2015). Amyloid β Protein and Alzheimer’s Disease:
When Computer Simulations Complement Experimental Studies. Chem. Rev.

[50] Rosenman D. J., Connors C. R., Chen W., Wang C., García A. E. (2013). Aβ
Monomers Transiently Sample Oligomer and Fibril-Like Configurations:
Ensemble Characterization Using a Combined MD/NMR Approach. J. Mol. Biol.

[51] Krupa P., Quoc Huy P. D., Li M. S. (2019). Properties
of Monomeric Aβ42
Probed by Different Sampling Methods and Force Fields: Role of Energy
Components. J. Chem. Phys.

[52] Nguyen P. H., Ramamoorthy A., Sahoo B. R., Zheng J., Faller P., Straub J. E., Dominguez L., Shea J.-E., Dokholyan N. V., De Simone A., Ma B., Nussinov R., Najafi S., Ngo S. T., Loquet A., Chiricotto M., Ganguly P., McCarty J., Li M. S., Hall C., Wang Y., Miller Y., Melchionna S., Habenstein B., Timr S., Chen J., Hnath B., Strodel B., Kayed R., Lesné S., Wei G., Sterpone F., Doig A. J., Derreumaux P. (2021). Amyloid Oligomers:
A Joint Experimental/Computational Perspective on Alzheimer’s
Disease, Parkinson’s Disease, Type II Diabetes, and Amyotrophic
Lateral Sclerosis. Chem. Rev.

[53] Wood S. J., Wetzel R., Martin J. D., Hurle M. R. (1995). Prolines and Aamyloidogenicity
in Fragments of the Alzheimer’s Peptide.Beta./A4. Biochemistry.

[54] Nerelius C., Sandegren A., Sargsyan H., Raunak R., Leijonmarck H., Chatterjee U., Fisahn A., Imarisio S., Lomas D. A., Crowther D. C., Strömberg R., Johansson J. (2009). α-Helix
Targeting Reduces Amyloid-β Peptide Toxicity. Proc. Natl. Acad. Sci. U. S. A.

[55] Saravanan K. M., Zhang H., Zhang H., Xi W., Wei Y. (2020). On the Conformational
Dynamics of β-Amyloid Forming Peptides: A Computational Perspective. Front. Bioeng. Biotechnol.

[56] Shi J., Zhang L., Liu E. (2017). Dissecting the Behaviour of Β-amyloid
Peptide Variants during Oligomerization and Fibrillation. J. Pept. Sci.

[57] Khaled M., Rönnbäck I., Ilag L. L., Gräslund A., Strodel B., Österlund N. (2023). A Hairpin
Motif in the Amyloid-β
Peptide Is Important for Formation of Disease-Related Oligomers. J. Am. Chem. Soc.

[58] Schäffler M., Wales D. J., Strodel B. (2024). The Energy
Landscape of Aβ_42_: A Funnel to Disorder for the Monomer
Becomes a Folding
Funnel for Self-Assembly. Chem. Commun.

[59] Yang M., Teplow D. B. (2008). Amyloid β-Protein
Monomer Folding: Free-Energy
Surfaces Reveal Alloform-Specific Differences. J. Mol. Biol.

[60] Mithu V. S., Sarkar B., Bhowmik D., Das A. K., Chandrakesan M., Maiti S., Madhu P. K. (2014). Curcumin Alters
the Salt Bridge-Containing
Turn Region in Amyloid β(1–42) Aggregates. J. Biol. Chem.

[61] Petkova A. T., Leapman R. D., Guo Z., Yau W.-M., Mattson M. P., Tycko R. S.-P. (2005). Molecular-Level
Polymorphism in Alzheimer’s
ß-Amyloid Fibrils. Science.

[62] Reddy G., Straub J. E., Thirumalai D. (2009). Influence of Preformed Asp23–Lys28
Salt Bridge on the Conformational Fluctuations of Monomers and Dimers
of Aβ Peptides with Implications for Rates of Fibril Formation. J. Phys. Chem. B.

[63] Tarus B., Straub J. E., Thirumalai D. (2006). Dynamics of
Asp23–Lys28 Salt-Bridge
Formation in Aβ_10–35_ Monomers. J. Am. Chem. Soc.

[64] Schütz A. K., Vagt T., Huber M., Ovchinnikova O. Y., Cadalbert R., Wall J., Güntert P., Böckmann A., Glockshuber R., Meier B. H. (2015). Atomic-Resolution
Three-Dimensional Structure of Amyloid β Fibrils Bearing the
Osaka Mutation. Angew. Chem., Int. Ed.

[65] Yang Y., Arseni D., Zhang W., Huang M., Lövestam S., Schweighauser M., Kotecha A., Murzin A. G., Peak-Chew S. Y., Macdonald J., Lavenir I., Garringer H. J., Gelpi E., Newell K. L., Kovacs G. G., Vidal R., Ghetti B., Ryskeldi-Falcon B., Scheres S. H. W., Goedert M. (2022). Cryo-EM Structures
of Amyloid-β 42 Filaments from Human Brains. Science.

[66] Colvin M. T., Silvers R., Ni Q. Z., Can T. V., Sergeyev I., Rosay M., Donovan K. J., Michael B., Wall J., Linse S., Griffin R. G. (2016). Atomic
Resolution Structure of Monomorphic
Aβ_42_ Amyloid Fibrils. J. Am.
Chem. Soc.

